# Impacts of Mechanical Injury on Volatile Emission Rate and Composition in 45 Subtropical Woody Broad-Leaved Storage and Non-Storage Emitters

**DOI:** 10.3390/plants14050821

**Published:** 2025-03-06

**Authors:** Yali Yuan, Yimiao Mao, Hao Yuan, Ming Guo, Guomo Zhou, Ülo Niinemets, Zhihong Sun

**Affiliations:** 1 College of Agriculture and Biology, Liaocheng University, Liaocheng 252000, China; yuanyl232323@163.com; 2 College of Horticultural Science, Zhejiang A&F University, Hangzhou 311300, China; 15136569982@163.com (Y.M.); yuanhao20220406@163.com (H.Y.); 3 College of Chemistry and Materials Engineering, Zhejiang A&F University, Hangzhou 311300, China; guoming@zafu.edu.cn; 4 School of Environmental and Resources Science, Zhejiang A&F University, Hangzhou 311300, China; zhougm@zafu.edu.cn; 5 Institute of Agricultural and Environmental Sciences, Estonian University of Life Sciences, 51006 Tartu, Estonia

**Keywords:** terpenoids, aromatics, mechanical injury, stress tolerance, oil cells, glandular trichomes, leaf longevity, idioblasts

## Abstract

Biogenic volatile organic compounds (BVOCs) significantly impact air quality and climate. Mechanical injury is a common stressor affecting plants in both natural and urban environments, and it has potentially large influences on BVOC emissions. However, the interspecific variability in wounding-induced BVOC emissions remains poorly understood, particularly for subtropical trees and shrubs. In this study, we investigated the effects of controlled mechanical injury on isoprenoid and aromatic compound emissions in a taxonomically diverse set of 45 subtropical broad-leaved woody species, 26 species without and in 19 species with BVOC storage structures (oil glands, resin ducts and glandular trichomes for volatile compound storage). Emissions of light-weight non-stored isoprene and monoterpenes and aromatic compounds in non-storage species showed moderate and variable emission increases after mechanical injury, likely reflecting the wounding impacts on leaf physiology. In storage species, mechanical injury triggered a substantial release of monoterpenes and aromatic compounds due to the rupture of storage structures. Across species, the proportion of monoterpenes in total emissions increased from 40.9% to 85.4% after mechanical injury, with 32.2% of this increase attributed to newly released compounds not detected in emissions from intact leaves. Sesquiterpene emissions, in contrast, were generally low and decreased after mechanical injury. Furthermore, wounding responses varied among plant functional groups, with evergreen species and those adapted to high temperatures and shade exhibiting stronger damage-induced BVOC emissions than deciduous species and those adapted to dry or cold environments. These findings suggest that mechanical disturbances such as pruning can significantly enhance BVOC emissions in subtropical urban forests and should be considered when modeling BVOC fluxes in both natural and managed ecosystems. Further research is needed to elucidate the relationship between storage structure characteristics and BVOC emissions, as well as their broader ecological and atmospheric implications.

## 1. Introduction

Plant-produced biogenic volatile organic compounds (BVOCs) play crucial roles in plant growth, reproduction, defense and stress responses [1,2,3]. They also significantly influence atmospheric chemistry and climate by affecting ozone and particulate matter formation [4,5,6]. Predicting the source strength of vegetation BVOC release and impacts of vegetation on atmospheric processes requires detailed information of the capacity of individual species to produce BVOC, as well as the stress responses of BVOC emissions. The East-Asian subtropics, with its high species diversity, is predicted to be a major source of global BVOC emissions [7,8,9]. However, the information on BVOC emissions and their stress responses in this region remains limited [10].

Based on the temporal variation of emissions and stress responses of emissions, BVOC emissions can be divided into constitutive and induced emissions. Constitutive BVOC production occurs in both stressed and unstressed conditions, whereas only certain species are strong constitutive emitters [2,11,12]. Induced BVOC emissions are elicited by stress, including biological stresses like pathogen infection and herbivory and abiotic stresses such as heat or mechanical injury [11,13,14]. Volatile isoprenoids, including isoprene, monoterpenes and sesquiterpenes, are the most abundant BVOCs emitted by plants [2,15,16]. Isoprene, a C5 hemiterpene, is constitutively emitted at high rates from some widespread temperate and subtropical and tropical woody species and from a few herbaceous species [10,17,18,19]. Monoterpenes (C10) are mainly emitted constitutively in evergreen conifers, as well as in several widespread evergreen and deciduous broad-leaved species [20,21]. Compared with monoterpenes, the constitutive emissions of sesquiterpenes (C15) are generally lower across emitting species and mainly reflect stress-induced emissions; they are typically emitted as part of a complex blend of stress-induced volatiles [11,22]. Volatile aromatics, including many benzenoids, form another major class of emitted BVOCs that play key roles in reproduction and physiological stress responses [11,23].

Upon production, volatiles can be immediately released to the atmosphere or stored in specialized storage structures [11,24,25]. Isoprene, due to its chemical properties, cannot be stored within leaves, whereas larger isoprenoids and aromatics may either be released immediately or stored, depending on the species, site of compound synthesis, and environmental stimuli [23,26]. Only species with specialized storage structures such as idioblasts, oil glands, glandular trichomes and resin ducts can store volatile isoprenoids and benzenoids in large quantities [21]. BVOC emissions vary in dependence on short- and long-term alterations in environmental conditions and in response to biotic and abiotic stresses [1,26]. In non-storage species, stress effects are modulated by changes in substrate availability for compound synthesis and gene expression levels that control the induction of new compounds [27,28]. In storage species with intact storage structures, the volatiles can leak out from the storage at a low rate, but the emissions can increase massively when these structures are mechanically damaged by herbivores or mechanical stress generated, e.g., by wind [29,30,31].

Mechanical injury, a common stressor in both natural and managed ecosystems (especially through pruning in urban areas), triggers both immediate and longer-term plant responses [31]. Immediate responses include the release of stored volatiles due to storage structure breakage, along with production of methanol and green leaf volatiles (GLVs) at the sites of damage [31,32,33]. These emissions can expel herbivores and attract their natural enemies [32] and possess antimicrobial properties [34]. Wounding can also reduce photosynthesis and thus affect the release of non-stored volatiles like isoprene [31]. Longer-term responses involve changes in substrate availability and gene expression related to BVOC synthesis, leading to altered emission rates and changed emission blends [35,36,37].

Although the importance of wounding on BVOC emissions is recognized, comparative studies across species, particularly in the East-Asian subtropics, are limited [31,33,38]. We hypothesize that (1) mechanical-injury-induced changes in BVOC emission rates and composition will vary significantly among species; (2) these variations will be related to the presence and characteristics of storage structures; and (3) species’ ecological adaptations (e.g., resistance to drought) will influence their wounding responses. Specifically, we predict that species adapted to moist, low-light conditions will have well-developed storage structures and show a rapid burst of diverse emissions upon mechanical injury, while species adapted to dry, high-light conditions will have reduced storage capacity and a less pronounced emission response. To test these hypotheses, we investigated the effects of mechanical injury on BVOC emissions from a set of 45 diverse broad-leaved tree and shrub species commonly found in the northern subtropical region. The species were grouped according to presence of storage structures (with and without storage structures), leaf longevity (evergreen and deciduous), and ecological tolerance category. In this study, we focused on isoprenoid (especially monoterpenes and sesquiterpenes) and aromatic compound emissions and report GLV emissions in a separate paper. Our previous work [11,39,40] has shown a correlation between constitutive BVOC emissions and species’ adaptations to light availability and drought. This study extends that work by examining the impact of wounding, a key stressor in both natural and urban environments.

## 2. Results

### 2.1. Impacts of Mechanical Injury on BVOC Emission Rates as Affected by the Presence of Storage Structures

#### 2.1.1. Isoprene Emissions

**Figure 1:**
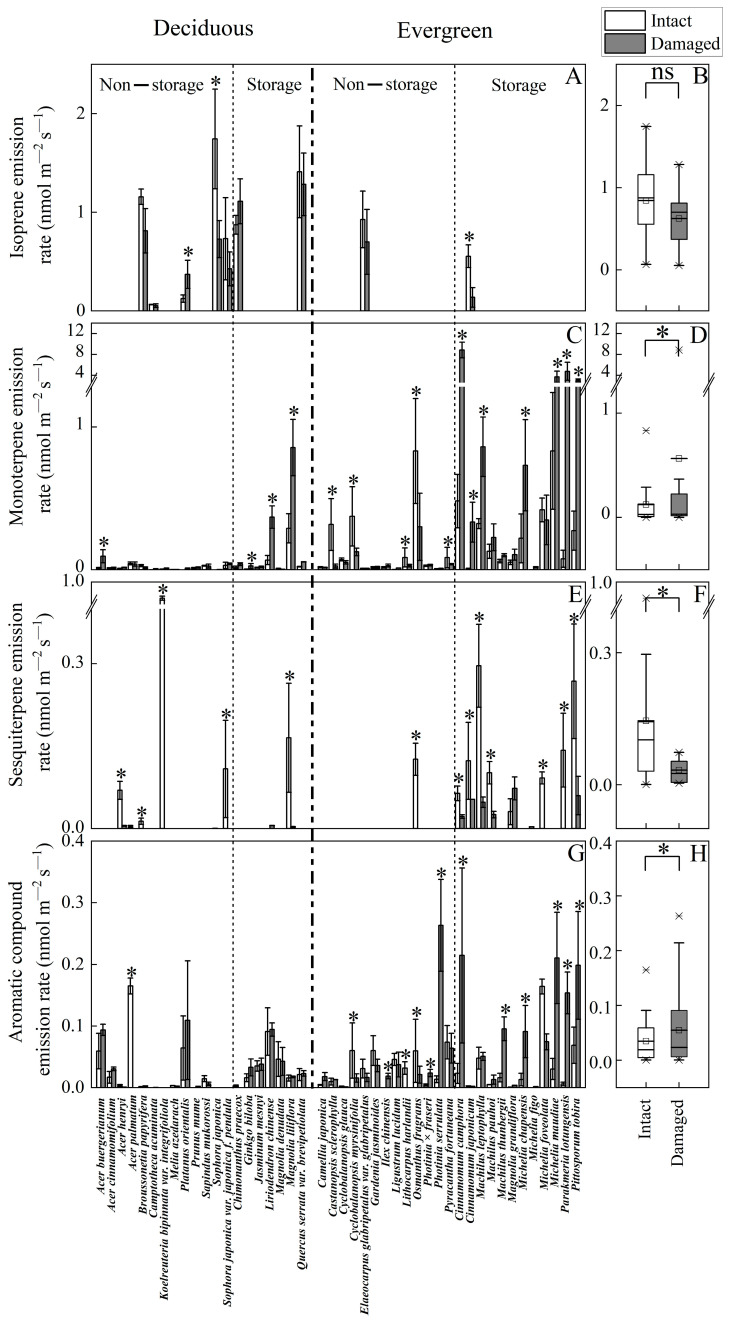
Emission rates of isoprene (**A**,**B**), monoterpenes (**C**,**D**), sesquiterpenes (**E**,**F**) and aromatic compounds (**G**,**H**) from intact and mechanically injured leaves of 45 subtropical tree species (Table 1) classified among deciduous and evergreen species and species having or lacking specialized volatile-storing structures (resin ducts, glandular trichomes, oil cells and oil glands). (**A**,**C**,**E**,**G**) are the species actual average (±SE) emission rates, and (**B**,**D**,**F**,**H**) are the mean emission rates for intact and damaged leaves. Three replicate trees were sampled for each species, and from each tree, two leaves were measured. The values for two replicate leaves per plant were averaged and then the average for three replicate plants was calculated. * denotes significant differences between intact and injured leaf blade emission rates according to paired-samples *t*-tests (*p* < 0.05). “ns” denotes no significant difference between the emission rates of intact and injured leaf blade.

Mechanical injury differently altered the emission rate depending on compound class and species (Figure 1, Table 1 and Table 2, Appendix A). Isoprene was emitted in nine tree species, and its emission rate was moderately affected by mechanical injury with some species showing increases and others decreases. A negative impact of mechanical injury on isoprene emission was observed in *Sophora japonica* (on average ± SE reduction of 58.3 ± 7.5%, *p* < 0.05) and *Cinnamomum japonicum* (75.3 ± 13.3% reduction, *p* < 0.05; Figure 1A), while in Platanus orientalis there was a significant increase (Figure 1A, *p* < 0.05). Given the contrasting species and overall moderate responses, the mechanical injury impact for all species pooled was not statistically significant (*p* > 0.05 for paired samples *t*-test, Figure 1B).

#### 2.1.2. Monoterpene Emissions

Monoterpene emissions were detected in all 45 tree species, with the effects of mechanical injury varying based on storage structures and leaf longevity (Figure 1C, Table 2, Appendix A). Mechanical injury increased monoterpene emissions in several evergreen species with leaf storage structures: *Parakmeria lotungensis* (62-fold), *Cinnamomum camphora* (17-fold), *Pittosporum tobira* (10-fold), and *Michelia maudiae* (3.5-fold). Conversely, wounding decreased emissions in most evergreen species without storage tissues, such as Castanopsis sclerophylla (12.7-fold reduction), *Cyclobalanopsis myrsinifolia* (3.0-fold), *Lithocarpus harlandii* (31-fold), and *Osmanthus fragrans* (2.8-fold). In deciduous species, wounding impacts ranged from moderately positive to negligible, except for *Magnolia liliflora* and *Liriodendron chinense*, which have storage structures. Overall, monoterpene emissions significantly increased in species with storage structures, especially evergreens (Figure 1C).

#### 2.1.3. Sesquiterpene Emissions

Sesquiterpene emissions were detected in 20 species, predominantly in evergreen species with specialized storage structures, though the emission rates were generally low (<0.3 nmol m^−2^ s^−1^ for most species; Figure 1E,F, Table 2, Appendix A). Mechanical injury reduced sesquiterpene emissions in 16 species by an average of 3.4-fold compared to intact leaves. Post-injury, only nine woody species continued to emit detectable levels of sesquiterpenes, while the emissions fell below the detection limit in the remaining seven species, e.g., in *Koelreuteria bipinnata* var. *Integrifoliola* from 0.86 ± 0.02 nmol m^−2^ s^−1^ and *Sophora japonica* var. *japonica f. pendula* from 0.108 ± 0.08 nmol m^−2^ s^−1^ to below the detection limit. Overall, sesquiterpene emissions were generally low and decreased after injury in most species.

#### 2.1.4. Aromatic Compound Emissions

Emissions of aromatic compounds from intact leaves were observed in 38 woody species, and wounding strongly stimulated these emissions, in particular in evergreen species with storage structures (Figure 1G, Table 2). For instance, after the injury, the emissions of aromatics increased 25-fold in *Parakmeria lotungensis*, 18-fold in *Photinia serrulata*, 8-fold in *Cinnamomum camphora*, and 6-fold in *Michelia maudiae* compared to the emissions from intact leaves (Figure 1G). In contrast, the emissions were reduced in several evergreen species without storage structures, including *Cyclobalanopsis myrsinifolia*, *Lithocarpus harlandii* and *Osmanthus fragrans*. In most deciduous species, the emission rates were only moderately affected by wounding (*p* < 0.05). For all species pooled, aromatic compound emissions significantly increased in species with storage structures. (Figure 1H, Table 2, Appendix A).

### 2.2. Impacts of Mechanical Injury on the BVOC Composition and Abundance in Storage and Non-Storage Species

**Figure 2:**
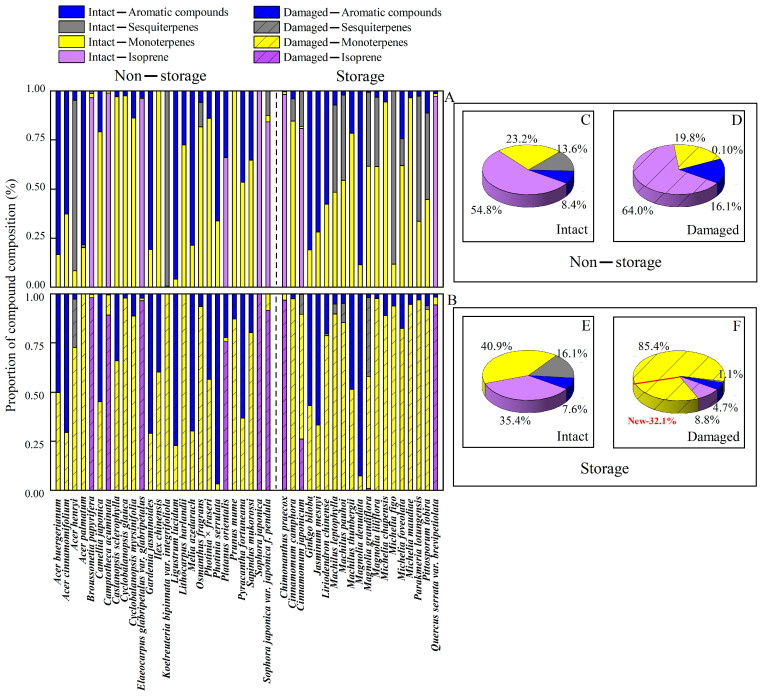
The proportions of compounds (monoterpenes, sesquiterpenes and aromatic compounds) in intact (**A**) and damaged (**B**) leaves in species with and without volatile storage structures and group-average share of BVOC classes for intact (**C**) and damaged (**D**) leaves in species without storage structures and for intact (**E**) and damaged (**F**) leaves in storage species. “New” represents the share of compounds not observed in the emissions of intact leaves and emitted after the leaves were injured.

Wounding altered the proportion of different BVOC compound classes and individual compounds in the emissions of 45 tree species, whereas the changes in BVOC composition depended on the presence of storage structures (Figure 1 and Figure 2, Table 2). In non-storage species, the share of isoprene (from 54.7% in intact leaves to 64.0% in wounded leaves) and aromatics (8.3% in intact vs. 16.1% in wounded leaves) increased upon wounding (Figure 2C,D). This was associated with reduced monoterpene (23.2% in intact vs. 19.8% in wounded leaves) and sesquiterpene (13.6% in intact vs. 0.10% in wounded leaves) emissions (Figure 2C,D). In storage species, the share of isoprene (from 35.5% in intact to 8.8% in wounded leaves), aromatics (7.6% in intact vs. 4.7% in wounded leaves), and sesquiterpenes (16.1% in intact vs. 1.0% in wounded (Figure 2E,F) decreased after mechanical injury. The monoterpene share strongly increased upon wounding (40.9% in intact vs. 85.4% wounded leaves) (Figure 2E,F).

#### 2.2.1. Injury-Dependent Changes in the Composition of Emitted Monoterpenes

Wounding altered the compound share within key volatile classes, especially for monoterpenes (Table 2, Appendix A). In species with storage structures, wounding enhanced common monoterpene emissions (e.g., α-pinene, α-terpinolene, limonene, camphor, cyclofeuchene, *β*-phellandrene, *α*-phellandrene, camphene, (*E*)-β-ocimene, *γ*-terpinene, pseudolimonene, *α*-terpinene, *p*-cymene, fenchene, *α*-thujene and eucalyptol, Appendix A). Furthermore, many new monoterpene compounds not emitted from intact leaves in the given species were induced after mechanical injury, including limonene in *Cinnamomum japonicum* and *Michelia figo*; camphor in *Magnolia grandiflora*, *M. liliflora*, *Michelia foveolata* and *Parakmeria lotungensis*; and cyclofeuchene in *C. camphora*, *P*. *lotungensis* and *Pittosporum tobira* (Table 2, Appendix A). Overall, 32.2% of wounding-dependent increase in the share of monoterpene emission was due to induction of new compounds. In storage species, a reduction or disappearance of emissions of certain monoterpenes was rare after wounding; such modifications were only observed in *Machilus pauhoi* (cyclofeuchene), *Machilus thunbergii* and *P. tobira* (*p*-cymene) and *Michelia foveolata* (α-pinene).

#### 2.2.2. Changes in Sesquiterpene Composition in Response to Mechanical Injury

Sesquiterpene diversity was overall low, and the main compounds observed in emissions from intact leaves were *β*-longipinene, *α*-muurolene, *β*-maaliene, *cis*-muurola-4(14),5-diene, *β*-caryophyllene and *α*-bulnesene (Table 2, Appendix A). The elicitation of new compounds after wounding was rare, with *cis*-muurola-3,5-diene observed in wounded leaves of *Liriodendron chinense*, *β*-bourbonene in *Magnolia denudata* and *β*-copaene in *M. grandiflora*. Consistent with the overall reduction in emission rate, the emission rate of most individual monoterpenes was reduced, and multiple sesquiterpenes were no longer detected in the emission blends (Table 2, Appendix A).

#### 2.2.3. Modifications in the Composition of Aromatics After Mechanical Injury

The change in composition of aromatic compounds after mechanical injury was different for storage and non-storage species (Table 2, Appendix A). 2, 5-dimethylstyrene was mainly emitted from evergreen species with storage structure belonging to the families *Lauraceae* and *Magnoliaceae*, and its emissions were significantly stimulated after wounding in these species. The compounds 2-phenylethyl formate and 3, 4-dimethylbenzyl alcohol were only observed in evergreens from *Lauraceae* and *Magnoliaceae*, e.g., in *Michelia chapensis*, *M. maudiae* and *Parakmeria lotungensis*.

In non-storage species, the responses of different aromatic compound emissions to wounding were variable. Emissions of 4-cyclopentyl ethylbenzoate, 2,5-dimethyl styrene, 2-phenylethyl benzoate and 2,4-butylethyl benzoate increased in *Camellia japonica*, *Ligustrum lucidum* and *Photinia serrulata*, while the emissions of these compounds decreased and reached even to levels below the detection limit in *Cyclobalanopsis myrsinifolia*, *Elaeocarpus glabripetalus* var. *glabripetalus* and *Osmanthus fragrans* (Table 2, Appendix A).

### 2.3. Response of BVOC Emission to Mechanical Injury in Relation to Leaf Longevity and Species Ecological Adaptations

#### 2.3.1. Changes in BVOC Emission Due to Mechanical Injury Associated with Leaf Longevity

**Figure 3:**
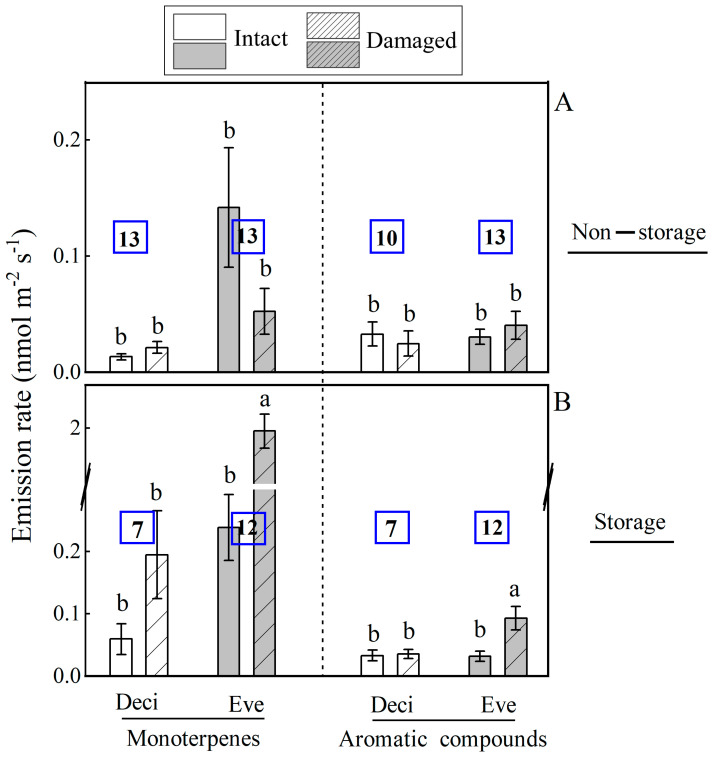
Comparison of average ± SE monoterpene and aromatic compound emission rates among intact and mechanically injured evergreen and deciduous leaves with (19 species) and without (26 species) specialized storage for 45 subtropical woody species. The number of species for each group is shown within each bar. Different letters denote significant differences (*p* < 0.05) between intact and injured leaf blades among the groups according to ANOVA followed by Duncan tests. The comparisons for isoprene and sesquiterpenes were not informative due to too few species for presence of storage/leaf duration combinations (*n* = 19). The abbreviations are Deci—deciduous species; Eve—evergreen species.

**Figure 4:**
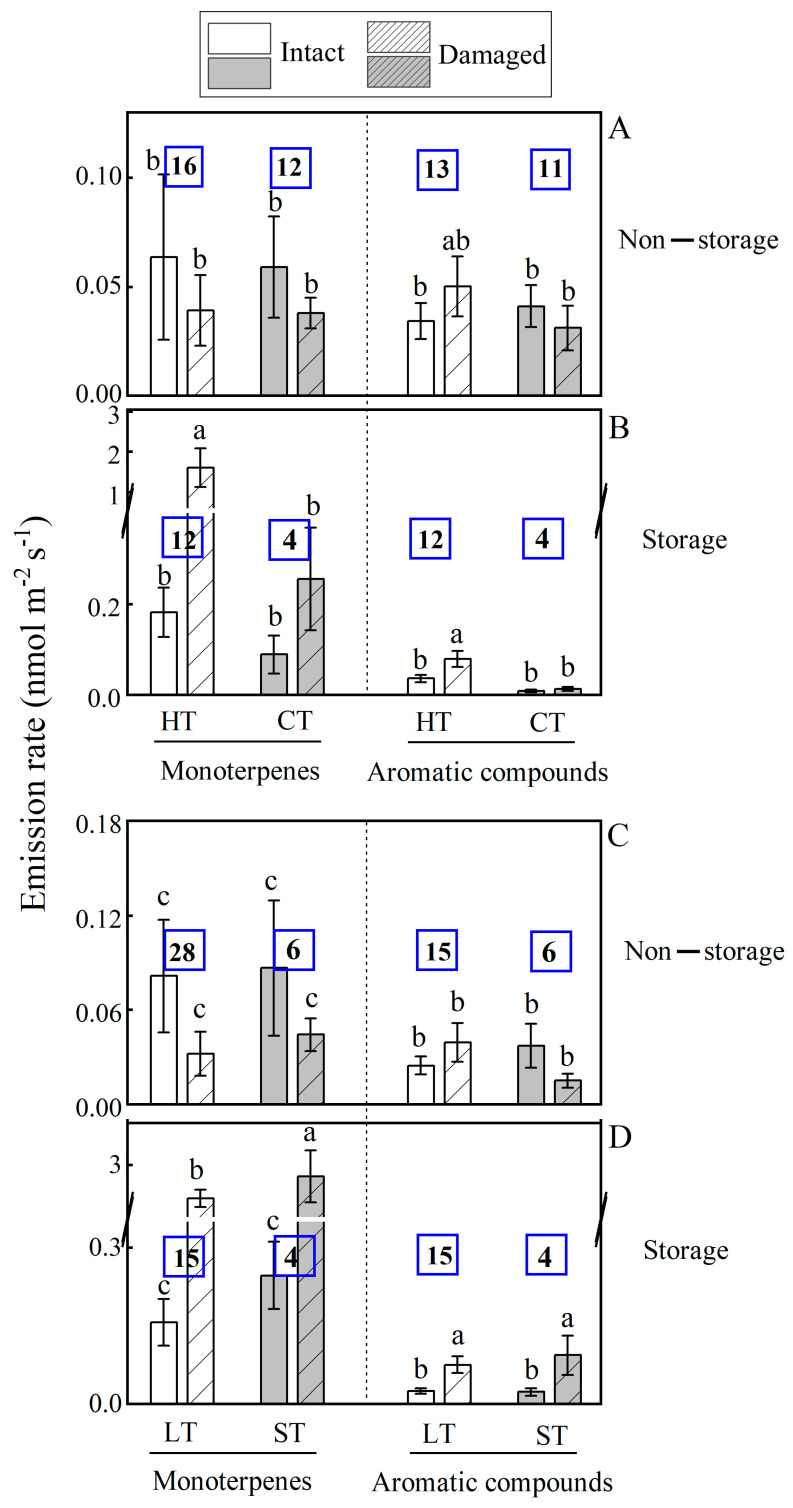
Comparison of average ± SE monoterpene and aromatic compound emission rates among intact and mechanically injured leaves of species with varying stress resistance, tolerant to high and low temperatures (**A**,**B**) and to high light and shade (**C**,**D**) among the 45 subtropical woody species studied. The number of species for each group is shown within each bar. Different letters denote significant differences (*p* < 0.05) between intact and injured leaves among the groups according to ANOVA followed by Duncan tests. Too few combinations of stress tolerance/presence of storage structure were available for isoprene and sesquiterpenes. Species ecological potentials are HT—high temperature resistant (thermophilus, *n* = 28); CT—cold tolerant (*n* = 16); LT—high-light resistant (*n* = 33); ST—shade tolerant (*n* = 11).

Mechanical injury altered BVOC composition and proportion among 45 woody species (Figure 1 and Figure 2), and the changes were related to leaf longevity and ecological tolerance (Figure 3 and Figure 4). Due to a limited number of isoprene- and sesquiterpene-emitting species, the impact of species ecology on wounding-dependent changes in emissions was analyzed for monoterpenes and aromatics. Storage species had strongly enhanced monoterpene emissions after mechanical injury, and non-storage species had unaffected emissions (Figure 3). Evergreen species generally had greater monoterpene emissions than deciduous woody species, and the wounding-dependent stimulation of monoterpene emission rate was greater in evergreen species (Figure 1C and Figure 3, Table 2, Appendix A). Aromatic compound emission was unaffected by wounding in deciduous species, and the emissions were enhanced in evergreens (Figure 3).

#### 2.3.2. Changes in BVOC Emission to Mechanical Injury in Species with Different Ecological Tolerance

The response of BVOC emission to mechanical injury was linked to the ecological tolerance of plant species, but differences in wounding responses among species were mainly due to variations in the proportion of storage species within each group (Figure 4). Wounding enhanced monoterpene emissions in storage species for all ecological groups analyzed (thermophilus species, HT; cold tolerant species, CT; high-light tolerant species, LT; shade tolerant species, ST), but wounding did not alter emissions in non-storage species for any of the ecological group. For aromatics, wounding increased the emissions in HT (Figure 4A,B), LT and ST (Figure 4C,D) species with storage structure and in none of the species groups without storage (Figure 4).

## 3. Discussion

Plants produce different volatile secondary metabolites (BVOC) as a defense strategy against different stresses. The volatiles can be immediately released after formation or stored in specialized storage structures and released upon the breakage of the storage structures. In this study, wounding responses of BVOC emissions from 45 widespread East-Asian subtropical woody species of contrasting volatile storage capacity and ecological requirements were investigated. Our study demonstrates major differences in the wounding responses among storage and non-storage species and among different volatile groups, isoprene, mono- and sesquiterpenes and aromatics, as discussed below.

### 3.1. Changes in Isoprene Emission in Response to Mechanical Injury

Isoprene, a major constitutively synthesized volatile organic compound, plays crucial role in enhancing plant protection against abiotic stress. Specifically, it improves thermotolerance [18,54,55] by maintaining thylakoid membrane integrity [56,57,58] and scavenging reactive oxygen species (ROS) [1,29,59,60]. Due to its high volatility, isoprene cannot be significantly stored within the leaves and is immediately released upon synthesis. Isoprene is synthesized in the chloroplasts from glyceraldehyde 3-phosphate and pyruvate via 2-C-methyl-D-erythritol-4-phosphate/ 1-deoxy-D-xylulose 5-phosphate (MEP/DOXP) pathway. Isoprene emission is closely associated with photosynthesis [61,62,63], which provides the necessary carbon substrates, ATP and NADPH [64,65,66,67]. However, differently from photosynthesis, moderately severe abiotic stresses typically stimulate isoprene emission [68,69]. The reduction in leaf stomatal conductance is typically one of the earliest responses to stress, limiting CO_2_ entry and thereby decreasing photosynthesis. The response of isoprene emission to CO_2_ concentration is a curve with an optimum; isoprene emissions decrease both at CO_2_ concentrations below and above the optimum. Therefore, a moderate decrease in stomatal conductance can initially stimulate isoprene emission [20,70]. However, with stress progression, decreases in isoprene emissions have been observed in many studies [63,69] due to reductions in the immediate isoprene substrate, dimethylallyl diphosphate (DMADP) concentration [71].

When plant leaves are wounded, their water conducting pathways are disrupted, reducing stomatal conductance. Consequently, this decreases photosynthesis and might alter carbon availability for DMADP formation [31,72]. The degree of reduction in conductance at the given level of wounding varies among species [31], suggesting that the response of isoprene emission rate to mechanical injury can also vary among species. In *Populus tremula* × *P*. *tremuloides*, moderate wounding did not affect isoprene emission, whereas in *Eucalyptus globulus*, isoprene emission was slightly stimulated [31]. Such contrasting responses were confirmed in our study, where significant negative effects of wounding were observed in two species and a positive effect of wounding in one species, and no significant wounding effects were observed in the remaining six species (Figure 1, Table 2, Appendix A). This evidence collectively indicates that in the short-term (minutes to a few hours), wounding effects on isoprene emission are expected to be only moderate, except for some species with stronger physiological responses to the given degree of wounding. However, mechanical injury can elicit enhanced expression of the isoprene synthase gene, thereby resulting in increased isoprene emissions in several hours to days after wounding [35]. Our study did not assess such longer-term wounding effects and so far, species differences in long-term wounding responses of isoprene emission have not been studied. Further research simultaneously assessing injury impacts on leaf photosynthetic characteristics, isoprene emission and DMADP pool size right after injury through recovery are needed to gain an insight into species differences in wounding responses.

### 3.2. Responses of Monoterpene Emissions to Mechanical Injury

Like isoprene, monoterpenes are synthesized via the MEP/DOXP pathway in plant plastids [73,74]. However, only a limited number of species are strong constitutive emitters of both isoprene and monoterpenes [75]. In our study, such a species was *Cinnamomum japonicum* (Figure 1). As mentioned in the Introduction, leaf monoterpene emissions can result from de novo synthesis in leaf mesophyll and immediate release (de novo emissions) or from release of monoterpenes synthesized and stored in special secretory structures such as idioblasts, oil glands, resin ducts and glandular trichomes (storage emissions). It is anticipated that de novo monoterpene emissions will respond to mechanical wounding similarly to isoprene emission, i.e., wounding is expected to lead to moderate positive or negative changes or no changes in emission rate depending on how wounding alters chloroplastic substrate availability for monoterpene synthesis. Indeed, among non-storage species in our study, mechanical injury enhanced de novo monoterpene emissions in two species (*Acer buergerianum* and *Camptotheca acuminata*), reduced in six species and did not significantly affect the emission rate in 18 species (Figure 1C,D). Both *Camptotheca acuminate* and *Platanus orientalis* are typically isoprene emitters, and the mechanical injury induced only a limited number of new monoterpene compounds in these species (Table 2, Appendix A). Furthermore, deciduous woody species without storage structures had generally a very low monoterpene emission rate either in intact or wounded leaves, whereas evergreen species without storage leaf structures had a higher monoterpene emission rate (Figure 3).

Terpene-storing species accumulate substantial amounts of terpenes in specialized storage structures, and breakage of these structures by herbivore feeding or mechanical damage leads to the release of stored compounds at high rates [30,31,76]. The release of stored compounds from wounded leaves plays crucial roles in direct and indirect defense, deterring herbivores and inhibiting the entry and growth of pathogenic microorganisms [77]. In our study, monoterpene emissions strongly increased after mechanical injury in 14 of 19 storage species, though the magnitude of responses differed among individual species (Figure 1C,D and Figure 3, Table 2, Appendix A). Particularly strong enhancement of emissions by wounding, 10- to more than 60-fold increase was observed in the evergreen tree species *Cinnamomum camphora*, *Parakmeria lotungensis* and *Pittosporum tobira* (Figure 1C, Table 2). In contrast, monoterpene emissions from the other five storage species (*Jasminum mesnyi*, *Magnolia denudate*, *Machilus pauhoi*, *Magnolia grandiflora* and *Michelia foveolata*) did not significantly respond to wounding. We propose that the interspecific variation in monoterpene baseline emissions and emission responses to wounding, reflects both genetic factors intrinsic to the species, including the number and regulation of expression of monoterpene synthase genes, overall activity of MEP/DOXP pathway and of course, the type, size and density of specialized storage structures. Further studies are warranted to elucidate the relationships of injury responses of monoterpene emissions with storage types, as well as with the number of storage structures per unit leaf area.

In this study, the emissions of *α*-pinene, limonene, *α*-terpinolene and camphor were most frequent, and the emissions of all these volatiles typically increased after leaf injury (Table 2, Appendix A). However, mechanical injury also altered the composition of emitted monoterpenes, and many new monoterpenes not observed in intact leaves were released in several woody species (Figure 1, Table 2, Appendix A). For instance, new oxygenated and non-oxygenated monoterpenes, like 3-carene, *α*-terpineol, endo-borneol, (*E*)-4-thujanol and fenchol, were detected in the emissions of *Cinnamomum camphora*, *Michelia maudiae* and *Parakmeria lotungensis* and several other woody species (Table 2). In *Pittosporum tobira*, even six new monoterpene compounds (camphor, *α*-phellandrene, (*E*)-*β*-ocimene, pseudolimonene, *α*-terpinene and *α*-fenchene) were detected after injury (Table 2). The increase in the proportion of oxygenated compounds may indicate oxidation processes occurring once these compounds are exposed to atmospheric conditions [78,79], although, the share of oxygenated terpenes can also increase in the case of de novo synthesized terpenes [80]. Furthermore, the storage species can have different types of oil glands and glandular trichomes with different BVOC composition [22,81,82,83]. Provided the possible differences in the abundance of different types of oil glands or glandular trichomes [81] and/or terpene permeability of cells lining the outer surface of storage structures, not all storage compartments might have contributed to the emitted terpene blend of non-injured leaves at a level exceeding the threshold for detection. Once injured, however, the emissions from these structures might have significantly contributed to the emission blend. *Pittosporum tobira* is a special case with both capitate glandular trichomes and oil canals, and it is plausible that the particularly strong enhancement of emissions and spectacular change of the emission blend after damage reflects additional sharing from a storage compartment that did not contribute to emissions in non-damaged leaves.

Apart from physical explanations, mechanical injury activates the plant defense system and leads to the elicitation of the expression of genes responsible for synthesis of monoterpenes, sesquiterpenes and green leaf volatiles [35,84,85]. For example, when *Pinus massoniana* was artificially damaged or attacked by *Dendrolimus punctatus* larvae, in addition to the increase in stored monoterpene emissions, emissions of many new compounds were induced [86]. Although full activation of gene expression is relatively time-consuming [87,88], enhanced expression of isoprenoid synthases might be observed already in minutes to a few hours after stress application [35]. After damage, several species started to emit (*E*)-*β*-ocimene, which is a classic stress-induced monoterpene [89], suggesting that induction of de novo terpene synthesis did occur. Furthermore, we also observed emissions of several new monoterpenes from injured leaves of non-storage species, suggesting that elicitation of gene-expression-level responses did contribute to the modification of the blend of monoterpenes emitted from the injured leaves.

The South-East Asian subtropics are usually characterized by high humidity and supraoptimal temperatures, often exceeding 35–40 °C. In this study, the selected 45 species are commonly found and grow well in Chinese subtropical to tropical regions, although not all of them originate from warm humid climates. It is well established that warm humid climates are typically conducive to the proliferation of pathogenic microorganisms. In this study, deciduous woody species without leaf storage structure were mainly sun-adapted species resistant to photoinhibition and occasional drought in highly exposed habitats, while the evergreen species without leaf storage structures were moderately shade-tolerant with leathery leaves that provide strong resistance to biotic stress and prevent microbial invasion. From an ecological perspective, these results may reflect the trade-off between synthesis of different protective chemicals in plants. Constitutive isoprene emission primarily serves to maintain high photosynthetic efficiency under stress, while constitutive monoterpene emissions not only protect photosynthesis from stress but also deter insect attacks.

### 3.3. Alterations in Sesquiterpene Emissions by Mechanical Injury

Sesquiterpenes are characteristic components of the volatile bouquets produced by many flowers, whereas the emission rate from non-stressed leaves is typically barely detectable [30]. This was confirmed in our study where the sesquiterpene emissions were generally low, except for *Koelreuteria bipinnata* var. *integrifoliola*, and the emissions above the detection limit were observed in only 20 species (Figure 1, Figure 2 and Figure 3, Table 1 and Table 2). Similarly to monoterpenes, sesquiterpenes can be stored in different storage structures [25,27,29], and thus, damage to these storage sites is expected to significantly enhance sesquiterpene emissions. Leaf injury by herbivores has been shown to strongly induce sesquiterpene emission [90]. In contrast, in this study, in addition to *Liriodendron chinense*, sesquiterpene emissions significantly decreased in other species, often to the level below the detection limits (Figure 1, Table 2). These results maybe reflect the difference in the nature of mechanical injury compared to herbivore-induced damage.

Furthermore, these puzzling differences between mono- and sesquiterpene wounding responses might reflect different physico-chemical characteristics in these compound groups. Sesquiterpenes are generally more reactive in the ambient atmosphere than monoterpenes and volatile aromatics; most reactive sesquiterpenes, although not all, have atmospheric lifetimes on the order of a few minute [91,92]. There also can be spatial separation of mono- and sesquiterpene synthesis, e.g., in different types of glandular trichomes [93,94]. Given that application of wounding took 0.5–1 h, part of the sesquiterpenes released after the breakage of storage structures might have become oxygenated, sealing the sites of damage and reducing subsequent emissions of sesquiterpenes. Previously, a reduction in emissions after terpene oxidation has been observed in wounded needles of *Pinus pinea* [78]. In addition, analyses of the essential oil composition of broad-leaved woody species indicate that stored sesquiterpenes generally comprise a relatively small fraction of total stored terpenes [79,95,96,97]. Thus, we suggest that sesquiterpene oxidation in combination with a much lower source strength of sesquiterpenes explains the reduction in sesquiterpene emissions after wounding in our study (Figure 1E,F, Table 2, Appendix B). Similarly to our study, wounding reduced the content of sesquiterpenes in the broad-leaved evergreen *Melaleuca alternifolia* [79,98].

### 3.4. Aromatic Compound Responses to Mechanical Injury

In plants, the chloroplastic shikimic acid pathway provides aromatic amino acids and precursors for a variety of secondary metabolites [99]. This pathway also facilitates the synthesis of multiple volatile compounds that can be either immediately emitted (de novo emissions) or stored prior to emission, akin to terpenes [31,61,100,101]. Although the terpene and phenolic compound storage structures can be spatially separated [25,102,103], we observed that mechanical injury responses of emissions of aromatic compounds broadly reflected the responses of monoterpene emissions to wounding. In the case of de novo emissions, aromatics emissions were moderately reduced in four species and enhanced in three species, whereas in storage species, the emissions were strongly enhanced in six species (Figure 1G,H, Figure 3). Interestingly, the aromatic compound 2, 5-dimethyl styrene was significantly stimulated after wounding only in those species with storage structures, including *Cinnamomum camphora*, *Magnolia grandiflora*, *Michelia chapensis*, *Michelia maudiae*, *Parakmeria lotungensis* and *Pittosporum tobira* (Table 2, Appendix A). This result indicates that the leakage of aromatics from leaf storage compartments was that low in intact leaves that certain aromatics were non-detectable until the storage tissues were broken.

Additionally, we identified several novel aromatic compounds that were released only after wounding, e.g., 2-phenylethyl formate and 3, 4-dimethylbenzyl alcohol in species with storage structures and 4-cyclopentyl ethylbenzoate in some species without storage structures (Table 2, Appendix A). As discussed above, stresses can alter BVOC emissions by changing gene expression patterns, as has also been observed for volatile phenolics [26,61,100,104], and the change in the emission composition can reflect plant systemic response to mechanical injury [37]. Although the time between wounding and the measurements was relatively short, it is plausible that in our study, the composition change was partly driven by the initial phase of the gene expression level response.

Emissions of aromatics and monoterpenes from intact and wounded leaves were positively correlated for storage species (Table 3), indicating that the release of these compounds after wounding primarily reflects the breakage of storage structures. In non-storage species, there was also evidence of a moderately strong positive correlation between monoterpene and benzenoid release after damage (Table 3). As the synthesis of aromatics and monoterpenes occurs in plastids, this correlation indicates that both the MEP/DOXP and shikimate acid pathway responded similarly to the wounding stress in our study. However, in other studies, stress-dependent emissions of de novo released monoterpenes and aromatics were not strongly correlated [105] or were even negatively correlated [33,106]. Our study demonstrates that wounding of storage species does significantly contribute to the enhancement of BVOC emissions in vegetation and suggests that more work is needed to gain an insight into the cooperativity of different BVOC synthesis pathways under stress in non-storage species.

### 3.5. Effects of Plant Functional Types and Different Ecological Requirements on Species Emission After Mechanical Injury

Urban woody floras are comprised of species that belong to diverse plant functional types and exhibit varying ecological tolerances. In this study, average monoterpene emissions were enhanced by wounding both in deciduous and evergreen plant functional types, but the enhancement was greater for evergreens, especially for those with storage structures (Figure 1 and Figure 4). This reflected the more widespread occurrence of storage structures in evergreen than in deciduous species, and these differences are consistent with the so-called leaf economics spectrum that embraces the life strategy of deciduous and evergreen species [107]. The deciduous species typically have higher investment in photosynthetic machinery and lower investments in structural and chemical defenses, whereas longer-living leaves in evergreens have lower photosynthetic capacity and greater investments in more robust leaf structure and chemical defenses to protect the leaves from abiotic and biotic stresses [107].

Regarding species ecological requirements, high-temperature and high-light resistance, shade tolerance and drought tolerance were associated with a stronger wounding response in monoterpene and aromatics emissions (Figure 4). This is consistent with the predictions from the leaf economics spectrum that plants in more stressful environments should have higher defense investments to enhance leaf longevity [15,107,108]. As noted above, plants are exposed to particularly strong pressure of pathogenic microorganisms in warm humid subtropical and tropical climates. The thirteen deciduous woody species without storage structure primarily emitted isoprene (Appendix A); these species are primarily high-light-adapted species that also moderately tolerate drought. Thirteen evergreen species without storage structures were constitutive emitters of monoterpenes; these species were moderately shade-tolerant (Figure 1 and Figure 3). Notably, almost all of these evergreen species without storage structures originated from the subtropical regions of China, indicating these species have adapted to high-temperature and high-humidity climates. In such conditions their robust leaf structure provides strong resistance to biotic stress and prevents microbial invasion, and we argue that the emissions of monoterpenes contribute to the pathogen resistance in these species.

Nineteen species had specialized storage structures and with the exception for *Chimonanthus praecox* and *Jasminum mesnyi*, 5 deciduous species and 12 evergreen species originated from subtropics, and most of these species were shade-tolerance. These species exhibited significant emissions of monoterpenes and aromatic compounds, especially following mechanical damage. This suggests that the release of monoterpenes and aromatics plays a crucial role in maintaining leaf resistance to environments rich in pathogenic microorganisms.

**Figure 5:**
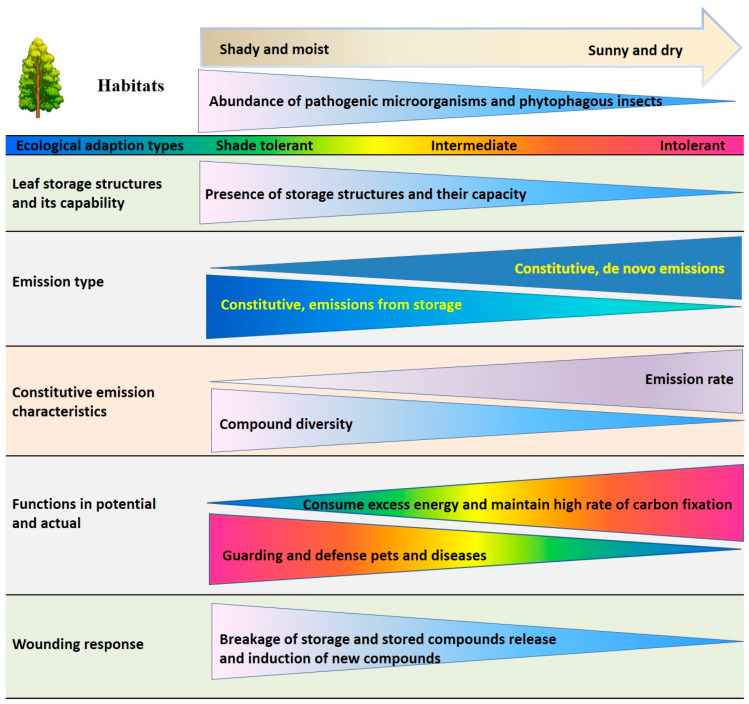
Schematic overview of emission characteristics of volatile isoprenoids and aromatics, the wounding responses of emissions, and volatile functions as associated with presence of leaf storage structures and ecological adaptations. The direction from the base to the vertex of the isosceles triangles indicates the change in the expression of the given trait.

From an adaptation perspective, the interspecific variations in emissions of isoprenoids and aromatics from intact leaves and after wounding may reflect the trade-offs in how plants invest energy and carbon to adapt to the variable environments. We hypothesize that constitutive isoprene emissions primarily serves to maintain high photosynthetic efficiency, in contrast, constitutive light-dependent monoterpene emissions not only support photosynthesis but also deter insect attacks. However, the stored monoterpenes and aromatics serve dual functions: they protect against herbivores in intact leaves and facilitate wound repair and healing upon mechanical injury. These distinct emission roles are closely associated with the presence or absence of leaf storage structures that reflect convergent evolution to enhance resistance to biotic stresses but also play an important role in plant survival in different environments (environmental tolerance; Figure 5).

## 4. Materials and Methods

### 4.1. Plant Selection and Sampling

The study was conducted in the campus of the Zhejiang Agriculture and Forestry University (30°15′23″ N, 119°43′29″ E, elevation 100–110 m). Firstly, 45 common ornamental woody species (25 evergreen and 20 deciduous species) were selected for the analysis. Based on species ecological characteristics, the selected species were classified as: high-temperature resistant (thermophilus species, MT, *n* = 28), cold tolerant (CT, *n* = 16), drought tolerant (DT, *n* = 13), waterlogging tolerant (WT, *n* = 5), high-light resistant (LT, *n* = 33) and shade tolerant (ST, *n* = 11) (Table 1) [10].

Sampling and BVOC emission measurements were conducted in summer (June–July) 2017, as described in detail in Yuan et al. (2020) [10]. For each species, three healthy adult individuals were selected. From each individual, two terminal branches (100–110 cm length) with mature leaves exposed to full sunlight were excised, immediately recut under water to avoid cavitation and transported to the laboratory for measurements.

### 4.2. Experimental Treatments

Two sets of BVOC measurements were conducted, one with intact branches and the other with mechanically damaged branches, as described in the next section. After the measurement of intact leaves, the leaves were subject to mechanical injury using a custom-made square (40 mm × 40 mm) hole punch with sharp micro-needles (88 needles uniformly distributed over the square surface). In each branch, all the leaves used for measurements of BVOC emissions were pierced, avoiding the leaf midrib. Once the mechanical injury was completed, typically in 30–40 min, BVOC release was immediately measured. Both for the control and damage treatment, 6 samples from 3 different plants (3 different plants × 2 branches) were measured for each species. During BVOC sampling, the chamber was illuminated with cold white LEDs, resulting in a light intensity at the leaf surface of 300 μmol m^−2^ s^−1^. This intensity corresponds to a mid-canopy light intensity in sunny days or above-canopy light intensity on cloudy days. It is typically not saturating for light-dependent BVOC emissions, but it was selected to avoid an excessive rise in temperature during sampling (leaf temperature during sampling was 27–29 °C). We note that the emission rates can be potentially scaled to standard conditions typically used to define the basal emission factor (incident light intensity of 1000 μmol m^−2^ s^−1^ and leaf temperature of 30 °C) [69]. The details are described in Yuan et al. (2020) [10].

### 4.3. Compound Sampling and Analysis

To increase the detection limit for low-level emitter and enhance the accuracy of detection of less dominant volatile compounds, a semi-closed dynamic sampling system was used, allowing the achievement of moderately high concentrations of volatiles during sampling. For BVOC sampling, the branches were enclosed in custom-made cylindrical glass (95% quartz glass) containers (either 15 cm × 25 cm or 10 cm × 15 cm, depending on the branch size). Extended glass tubes (0.8 cm diameter) were attached to the containers for BVOC sampling and ambient air entry. The effect of diffusion of BVOC-enriched air out of the container through the air inlet was estimated to affect the calculated BVOC emission rates <1% [10].

BVOC were collected with an air sampling pump (Beijing Municipal Institute of Labour Protection, Beijing, China) onto the adsorption tubes filled with Tenax TA, Carbograph TD and Carboxen 1003 (Markes, Bridgend, UK) at 120 mL·min^−1^ for 40 min. Further details of sampling are provided in Yuan et al. (2020). Volatile analysis was conducted with a gas chromatograph (GC; 7890B, Agilent, Santa Clara, CA, USA) equipped with a mass spectrometric (MS) detector (5977B, Agilent, Santa Clara, CA, USA) and thermal desorption (TD) system (TD-100, Markes, Bridgend, UK). A DB-624 (length 60 m, inner diameter 0.25 mm, film thickness 0.25 μm; Agilent, Santa Clara, CA, USA) capillary column was used. The MS was operated in electron impact (EI) ionization (70 eV) mode using the mass scan range of *m*/*z* 45–400. The ion source and transfer line temperature was 230 °C, the quadrupole temperature was 150 °C, and the flow rate of the carrier gas (He) was 1 mL min^−1^ for desorption and 1.2 mL min^−1^ for GC-MS analysis. The GC oven program used was as in [10].

Authentic GC-grade standards (Sigma, St. Louis, MO, USA) and MassHunter software (version B.07.00, Agilent, Santa Clara, CA, USA) with the NIST 14 spectral library were used to identify the compounds. To calibrate the system, a standard gas (Ionicon GmbH, Innsbruck, Austria) containing 15 different volatiles (each individual volatile at 1 ppm) was used and quantitatively diluted with high purity nitrogen (99.999%). The diluted calibration samples were collected onto the adsorption tubes and analyzed according to the same protocol as plant BVOC samples [10,98].

### 4.4. Determination of Retention Index and Compound Identification

To accurately identify biovolatile organic compounds (BVOCs), the retention index (RI) method combined with mass spectrometry data was employed to confirm the identity of the compounds. A series of known alkane standard substances (with carbon chain lengths ranging from C5 to C20, Sigma) were utilized to estimate the retention index of the test compound. By analyzing these standard substances, we obtained their respective retention times on the chromatographic column (*t_R_*_n_, *t_R_*_n+1_). Subsequently, BVOC samples were analyzed under identical chromatographic conditions as those used for the alkane standards, allowing us to record the retention time (*t_R_*_x_) for each target compound. The retention index (RI) was calculated using the following formula:

$$\mathrm{RI}=100\times \left(n+\frac{tR(x)-tR(n)}{tR\left(n+1\right)-tR(n)}\right)$$

n: number of carbon atoms in the alkane with a shorter retention time than that of the test compound; *t_R_*_x_: retention time of the test compound; *t_R_*_n_ and *t_R_*_n+1_: retention times of two alkanes with slightly shorter and longer retention times than that of the test compound, respectively.

The retention index of each BVOC calculated by the above method was then compared with the retention index data in the NIST 14 mass spectrometry database to quickly confirm the identity of the test compound, and the mass spectrometry data of each target compound were further confirmed. The detailed retention index data of each compound measured in this study are listed in Appendix B.

### 4.5. Classification and Identification of Leaf BVOC Storage Structures

As highlighted in the Introduction, the presence of BVOC storage structures can importantly alter the magnitude of terpene and aromatics emissions. Thus, the studied species were classified according to the presence of storage structures in leaves as non-storage and storage species (Table 1). The storage species were further classified according to the type of storage structures as species with oil cells, glandular trichomes or secretion (resin) ducts (Table 1). Out of 45 studied species, 15 species contained oil cells, including all *Lauraceae* species (*Cinnamomum camphora*, *Cinnamomum japonicum*, *Machilus leptophylla*, *Machilus pauhoi* and *Machilus thunbergii*) [45,46], *Magnoliaceae* species (*Magnolia denudata*, *Magnolia grandiflora*, *Magnolia liliflora*, *Michelia chapensis*, *Michelia figo*, *Michelia foveolata*, *Michelia maudiae*, *Parakmeria lotungensis* and *Liriodendron chinense*) [43,51] and a *Calycanthaceae* species (*Chimonanthus praecox*) [43]. Among the studied species, only *Ginkgo biloba* has resin ducts in leaves [47], and *Pittosporum tobira* has oil ducts [52].

Although the taxonomic signature for the presence of oil cells was strong, the association of glandular trichomes to taxonomy was weaker. Among the studied species, 4 species (*Cinnamomum camphora* from *Lauraceae*, *Jasminum mesnyi* from *Oleaceae*, *Pittosporum tobira* from *Pittosporaceae* and *Quercus serrata* var. *brevipetiolata* from *Fagaceae*) had glandular trichomes on the leaf surface [45,46,48,53]. In contrast, another species from *Fagaceae*, *Lithocarpus harlandii*, does not have glandular trichomes on the leaf surface [50]. On the other hand, *Koelreuteria bipinnata* var. *integrifoliola* has non-glandular trichomes and no glandular trichomes, so trichomes on the surface of this species do not play a role in the storage of volatiles [49]. In *Camptotheca acuminata* (*Nyssaceae*), glandular trichomes are found on the stem and on the surface of young leaves but they are not present on the surface of mature leaves [42]. Likewise, *Melia azedarach* (*Meliaceae*) has glandular trichomes on the surface of young but not on old leaves [109]. Therefore, these species were classified as non-storage species. Analogously, in most *Rosaceae* and *Oleaceae* species, the glandular trichomes are found on the flowers not on the leaves [41]. In *Fabaceae* and *Aceraceae* species, storage structures (oil cells) or glandular trichomes have not been found [41]. Altogether 19 species were confirmed to have at least one type of storage. As for some storage structure types, only one or a few species were available; the effect of storage structure type was assessed in the analyses of the impact of presence of storage structures on BVOC emissions (Table 1).

### 4.6. Statistical Analyses

All reported values are the averages of three independent replicates per species. First, BVOC emission rates were averaged for each plant individual (two replicate samples taken from each individual), and then the species average (three replicate plants) was calculated. The differences between the emission rates of intact and mechanically injured leaves were analyzed by paired sample *t*-tests. Differences in average emission rates before and after mechanical injury among species groups with and without storage structures, among deciduous and evergreen species and among ecological tolerance groups were analyzed by ANOVA followed by Duncan tests. Correlations between individual isoprenoid classes (isoprene, monoterpenes and sesquiterpenes) and aromatic compounds were explored by linear and nonlinear regression analyses. All statistical tests were considered significant at *p* < 0.05. The statistical analyses were performed with SPSS 24.0 for Windows (Statistical Products and Service Solutions, IBM, Armonk, NY, USA), and figures were drawn with Origin 8.0 software (Origin Lab, Northampton, MA, USA).

## 5. Conclusions

The presence and activity of different secondary metabolic pathways, including those responsible for BVOC production, are primarily governed by the intrinsic phylogenetic relationships and genetic backgrounds of the species. Nevertheless, even plant species with closely related genetic profiles have evolved distinct biological traits that align with their ecological niches. Wounding is a recurrent stress for plants, especially in urban environments where the vegetation is regularly pruned, but the information of interspecific variability in modifications in BVOC emission rates after wounding is scare. Our study emphasizes that the presence of storage structures is the key factor controlling the degree of wounding-dependent change in BVOC emissions to the ambient atmosphere. In particular, emissions of monoterpenes and aromatics in species with storage structures respond much more strongly to wounding than in species lacking storage structures. In the case of isoprene and de novo synthesized monoterpenes and aromatics in species without storage structures, the responses are moderate and their magnitude and direction likely depend on the impact of wounding on foliage photosynthetic characteristics that ultimately control the substrate availability for BVOC synthesis. Our study uncovered large differences in the wounding response among evergreen and deciduous species and among species with different tolerance of ecological factors. These differences were primarily driven by greater abundance of BVOC-storing species among evergreens and among species with higher tolerance of different abiotic stress factors. Evergreen and presence of storage structures reflect long-term plant adaptation to warm high-humidity environments, and furthermore, many evergreens can grow under low light conditions and have high shade tolerance. We argue that wounding-dependent stimulation of the emission of monoterpenes and aromatics need to be considered in emission inventories for urban forests. Our results can be applied in regional natural and urban emission inventories in the subtropical biome. Furthermore, the impact of wounding as an important factor in the broad-leaved evergreen forest biome has to be incorporated in global models. To better understand the modulation of BVOC emissions in response to wounding, further studies should explore the phylogenetic relationships in the presence of BVOC storage and ecological adaption traits and plant capacity to emit BVOC.

## Figures and Tables

**Table 1:** Information on leaf storage type, plant functional type (evergreen vs. deciduous) and species ecological characteristics for 45 subtropical woody broad-leaved species. “No” indicates that no storage compartment has been found in mature leaves. Species ecological characteristics are HT—high temperature resistant (thermophilus); CT—cold tolerant; LT—high-light resistant; ST—shade tolerant.

Number	Family	Species	Presence of Terpene-Storage Structures with Reference	Plant Functional Type	Species Ecological Characteristics
1	*Aceraceae*	*Acer buergerianum*	No [41]	Deciduous	HT; CT
2	*Aceraceae*	*Acer cinnamomifolium*	No [41]	Deciduous	HT; LT
3	*Aceraceae*	*Acer henryi*	No [41]	Deciduous	HT; CT
4	*Aceraceae*	*Acer palmatum*	No [41]	Deciduous	HT; CT; ST
5	*Moraceae*	*Broussonetia papyrifera*	No	Deciduous	LT
6	*Theaceae*	*Camellia japonica*	No	Evergreen	HT; ST
7	*Nyssaceae*	*Camptotheca acuminata*	No [42]	Deciduous	LT
8	*Fagaceae*	*Castanopsis sclerophylla*	No	Evergreen	LT
9	*Calycanthacecae*	*Chimonanthus praecox*	Oil cells [43]	Deciduous	CT; LT
10	*Lauraceae*	*Cinnamomum camphora*	Oil cells [44,45,46] and glandular trichomes [45,46]	Evergreen	HT; ST; LT
11	*Lauraceae*	*Cinnamomum japonicum*	Oil cells [44,45]	Evergreen	HT; LT
12	*Fagaceae*	*Cyclobalanopsis glauca*	No	Evergreen	CT; ST
13	*Fagaceae*	*Cyclobalanopsis myrsinifolia*	No	Evergreen	CT; ST
14	*Elaeocarpaceae*	*Elaeocarpus glabripetalus* var. *glabripetalus*	No	Evergreen	LT
15	*Rubiaceae*	*Gardenia jasminoides*	No [41]	Evergreen	HT
16	*Ginkgoaceae*	*Ginkgo biloba*	Resin ducts [47]	Deciduous	LT; CT
17	*Aquifoliaceae*	*Ilex chinensis*	No	Evergreen	HT; CT; ST
18	*Oleaceae*	*Jasminum mesnyi*	Glandular trichomes [48]	Deciduous	HT; ST; LT
19	*Sapindaceae*	*Koelreuteria bipinnata* var. *integrifoliola*	No [49]	Deciduous	HT; CT; LT
20	*Oleaceae*	*Ligustrum lucidum*	No	Evergreen	HT; CT; ST; LT
21	*Magnoliaceae*	*Liriodendron chinense*	Oil cells [43]	Deciduous	HT; LT
22	*Fagaceae*	*Lithocarpus harlandii*	No [50]	Evergreen	HT; LT
23	*Lauraceae*	*Machilus leptophylla*	Oil cells [44,45]	Evergreen	ST
24	*Lauraceae*	*Machilus pauhoi*	Oil cells [44,46]	Evergreen	ST
25	*Lauraceae*	*Machilus thunbergii*	Oil cells [44]	Evergreen	ST
26	*Magnoliaceae*	*Magnolia denudata*	Oil cells [43,44]	Deciduous	HT; LT
27	*Magnoliaceae*	*Magnolia grandiflora*	Oil cells [43,51]	Evergreen	HT; CT; LT
28	*Magnoliaceae*	*Magnolia liliflora*	Oil cells [43,51]	Deciduous	CT; LT
29	*Meliaceae*	*Melia azedarach*	No	Deciduous	HT; LT
30	*Magnoliaceae*	*Michelia chapensis*	Oil cells [43,51]	Evergreen	HT; LT
31	*Magnoliaceae*	*Michelia figo*	Oil cells [43]	Evergreen	HT; LT
32	*Magnoliaceae*	*Michelia foveolata*	Oil cells [43]	Evergreen	HT
33	*Magnoliaceae*	*Michelia maudiae*	Oil cells [43,51]	Evergreen	HT; LT
34	*Oleaceae*	*Osmanthus fragrans*	No [41]	Evergreen	HT; LT
35	*Magnoliaceae*	*Parakmeria lotungensis*	Oil cells [43,51]	Evergreen	HT; LT
36	*Rosaceae*	*Photinia* × *fraseri*	No	Evergreen	HT; LT
37	*Rosaceae*	*Photinia serrulata*	No [41]	Evergreen	HT; LT
38	*Pittosporaceae*	*Pittosporum tobira*	Oil ducts [52]	Evergreen	LT
39	*Platanaceae*	*Platanus orientalis*	No	Deciduous	HT; CT; LT
40	*Rosaceae*	*Prunus mume*	No [41]	Deciduous	CT; LT
41	*Rosaceae*	*Pyracantha fortuneana*	No	Evergreen	CT; LT
42	*Rosaceae*	*Quercus serrata* var. *brevipetiolata*	Glandular trichomes [53]	Deciduous	CT; LT
43	*Sapindaceae*	*Sapindus mukorossi*	No	Deciduous	LT
44	*Leguminosae*	*Sophora japonica*	No [41]	Deciduous	HT; LT
45	*Leguminosae*	*Sophora japonica* var. *japonica* f. *pendula*	No [41]	Deciduous	HT; LT

Note: The classification and identification of plant leaf storage structures is provided in “Materials and Methods”.

**Table 2:** Changes in emissions before and after mechanical injury in 45 East-Asian subtropical woody species. Depending on the change in compound emission rate, the compounds are classified among four categories: increased emission after injury; reduced emission after injury; compound disappearance (emission below detection limit) after injury; newly emitted compound after injury.

Compound			Changes in Compounds After Mechanical Injury								
			Plant Functional Type	Storage Species				Non-Storage Species			
				Increased	New Compound	Reduced	Disappeared	Increased	New Compound	Reduced	Disappeared
Monoterpenes	C_10_H_16_	*α*-pinene	Deciduous Evergreen	9, 16 *, 18, 21,26, 28, 42 * 10 *, 11 *, 23, 24 *, 30, 31 *, 33 *, 35 *, 38 *		27, 32		3, 4, 7, 29, 40, 43, 45; 14, 17 *, 20 *, 36	1; 34 *	5, 19; 6, 8 *, 12, 13, 22, 37, 41	
	C_10_H_16_	*α*-terpinolene	Deciduous Evergreen	9, 28; 10 *, 23, 24, 25, 30, 32 *, 33 *	38 *			12	1 *	13 *, 34 *	8 *, 22 *
	C_10_H_16_	limonene	Deciduous Evergreen	9 *, 21, 28 *, 42 * 10 *, 23 *, 24, 25 *, 27 *, 30, 32, 33 *, 35 *, 38 *	11 *, 31 *			12, 37	1 *, 7 *, 43 *, 6 *	8 *, 13, 34 *	3 *22 *
	C_10_H_16_O	camphor	Deciduous Evergreen	9 *, 16 *, 18, 26; 10 *, 33 *	28 *; 11 *, 27 *, 32 *, 35 *, 38 *	25		1, 2, 40, 42, 45; 12, 14, 15, 17, 36, 37	3 *, 7, 39 *; 34 *	4, 5, 29, 43; 6, 13, 22, 41	
	C_10_H_16_	cyclofeuchene	Deciduous Evergreen	33 *, 38 *	10 *, 35 *	23, 24				45 *; 8 *, 34 *	13 *
	C_10_H_16_	*β*-phellandrene	Deciduous Evergreen	9, 28; 23 *, 24, 30 *, 32 *, 33 *, 35 *	10 *, 11 *, 38 *						
	C_10_H_16_	*α*-phellandrene	Deciduous Evergreen	10 *, 23 *, 24, 30, 32 *, 33 *	21 *; 11 *, 35 *, 38 *				1 *		8 *, 22 *
	C_10_H_16_	camphene	Deciduous Evergreen	28; 11 *, 23, 24, 27 *, 30, 32 *, 33 *, 38 *	21 *; 10 *, 25 *, 31 *, 35 *			12, 41		13 *, 34 *	8 *, 36 *
	C_10_H_16_	(*E*)-*β*-ocimene	Deciduous Evergreen	28 *; 10 *, 33	9 *, 21 *; 11 *, 23 *, 24 *, 35 *, 38 *	32			36 *	22 *	2 *; 8 *, 41 *
	C_10_H_16_	*γ*-terpinene	Deciduous Evergreen	9 *, 21 *, 28 *; 10 *, 23, 24, 25 *, 27 *, 30 *, 32, 33 *	11 *, 35 *, 38 *						
	C_10_H_16_	pseudolimonene	Deciduous Evergreen	9, 21 *; 10 *, 23, 24, 27 *, 28, 30, 33 *, 35 *	11 *, 25 *, 38 *				1; 34 *, 42 *	13	8 *, 22 *
	C_10_H_16_	*α*-terpinene	Deciduous Evergreen	10 *, 23, 25 *, 27 *, 30, 32, 33 *	21 *, 28 *; 35 *, 38 *					13	8 *
	C_10_H_14_	*p*-cymene	Deciduous Evergreen	9 *, 28 *; 10 *, 11 *, 23 *, 27, 30, 33 *	21 *; 25 *, 35 *	24, 32	38 *			13 *, 34 *	8 *, 22 *
	C_10_H_16_	*α*-fenchene	Deciduous Evergreen	21; 10 *, 23, 30, 32 *	27 *, 35 *, 38 *						8 *, 13 *, 34 *
	C_10_H_16_	*α*-thujene	Deciduous Evergreen	28 *; 30 *, 32, 33 *		25	27 *				
	C_10_H_18_O	eucalyptol	Deciduous Evergreen	28; 10 *, 30, 32 *, 33 *, 35 *	21 *; 23 *						
	C_10_H_16_	3-carene	Deciduous Evergreen		21 *; 10 *, 30 *, 35 *, 38 *						
	C_10_H_16_	*β*-fenchene	Deciduous Evergreen	38 *	10 *, 23 *, 33 *, 35 *						
	C_10_H_18_O	*α*-terpineol	Deciduous Evergreen		28 *; 10 *, 33 *, 35 *						
	C_10_H_18_O	*trans*-4-thujanol	Deciduous Evergreen		10 *, 30 *, 33 *, 35 *						
	C_10_H_18_O	endo-borneol	Deciduous Evergreen		10 *, 11 *, 33 *, 35 *, 38 *						
	C_10_H_18_O	fenchol	Deciduous Evergreen		33 *, 35 *, 38 *						
Sesquiterpene	C_15_H_24_	*α*-bulnesene	Deciduous Evergreen			28; 11, 23, 27, 32	38 *				3 *
	C_15_H_24_	caryophyllene	Deciduous Evergreen				11 *, 23 *, 24 *, 27 *				
	C_15_H_24_	*β*-longipinene	Deciduous Evergreen				28 *; 10 *, 11 *, 23 *, 24 *, 32 *, 35 *, 38 *				19 *; 34 *
	C_15_H_24_	*γ*-humulene	Deciduous Evergreen				28 *; 10 *, 11 *, 32 *				
	C_15_H_24_	germacrene	Deciduous Evergreen				28 *; 23 *, 31 *				
	C_15_H_24_	*cis*-muurola-3,5-diene	Deciduous Evergreen		21 *						
	C_15_H_24_	*cis*-muurola-4(14),5-diene	Deciduous Evergreen				10 *, 11 *, 23 *, 27 *, 31 *, 38 *				4 *, 5 *, 44 *, 45 *; 8 *, 22 *
	C_15_H_24_	*β*-maaliene	Deciduous Evergreen				11 *, 23 *, 24 *, 32 *				
	C_15_H_24_	*α*-muurolene	Deciduous Evergreen				23 *, 24 *, 32 *				3 *, 45 *
	C_15_H_24_	bicyclogermacrene	Deciduous Evergreen				10 *, 11 *, 23 *, 27 *, 32 *, 38 *				3 *, 19 *
	C_15_H_24_	*β*-copaene	Deciduous Evergreen				11 *, 23 *, 32 *, 35 *, 38 *				19 *, 45 *
	C_15_H_24_	*δ*-guaiene	Deciduous Evergreen				38 *				3 *, 19 *
Aromatic compound	C_14_H_18_O_2_	4-cyclopentyl ethylbenzoate	Deciduous Evergreen	16 *, 18, 42		26; 10, 23, 32, 35 *	9 * 38 *	6 *, 20, 37	17 *	13 *, 15, 34 *	4 *; 22 *
	C_10_H_12_	2,5-dimethylstyrene	Deciduous Evergreen	28; 10 *, 23, 24, 27 *, 30 *, 33 *, 35 *, 38 *	21 *			1, 2 *		3 * 12, 34 *	8 *, 13 *, 22 *
	C_9_H_10_O_2_	2-phenylethyl formate	Deciduous Evergreen		25 *, 30 *, 32 *						
	C_9_H_12_O	3,4-dimethylbenzyl alcohol	Deciduous Evergreen		30 *, 33 *, 35 *						
	C_15_H_14_O_2_	2-phenylethyl benzoate	Deciduous Evergreen	18 *				5; 37 *	29 *, 40 * 12 *		14 *
	C_13_H_18_O_2_	2,4-butylethylbenzoate	Deciduous Evergreen						7 *; 8 *	43 *; 12, 14, 41	

* denotes significant differences between intact and injured leaf blade emission rates according to paired-samples *t*-tests (*p* < 0.05).

**Table 3:** Correlations between emission rates of isoprene and monoterpenes, sesquiterpenes and aromatics for intact and mechanically injured evergreen and deciduous leaves with (19 species) and without (26 species) specialized storage. Due to insufficient numbers of evergreen species emitting isoprene and damaged deciduous species emitting sesquiterpenes (*n* < 9), only those species that were sufficient are shown in the following table. “*n*” represents 3 repeated data for each tree species.

Compound Class	Presence of Storage Structures	Treatment	Isoprene		Monoterpenes				Sesquiterpenes		
			Deciduous		Deciduous		Evergreen		Deciduous	Evergreen	
			Intact	Damaged	Intact	Damaged	Intact	Damaged	Intact	Intact	Damaged
Monoterpenes	Non-storage	Intact	*r* = 0.656 *, *n* = 9	*r* = 0.805 *, *n* = 9	——	*r* = 0.265, *n* = 33	——	*r* = 0.813 **, *n* = 39	*r* = −0.185, *n* = 18	*n* = 3	*n* = 3
		Damaged	*r* = −0.237, *n* = 15	*r* = −0.297, *n* = 15	——	——	——	——	*n* = 3	——	——
	Storage	Intact	*n* = 3	*n* = 3	——	*r* = 0.807 *, *n* = 18	——	*r* = 0.671 *, *n* = 36	*r* = 0.695 *, *n* = 9	*r* = 0.606 *, *n* = 27	*r* = −0.097, *n* = 18
		Damaged	*n* = 3	*n* = 3	——	——	——	——	n = 6	*r* = 0.475, *n* = 27	*r* = −0.350, *n* = 18
Sesquiterpenes	Non-storage	Intact	*r* = −0.792 *, *n* = 9	*r* = 0.149, *n* = 9	*r* = −0.156, *n* = 15	*r* = −0.240, *n* = 18	*n* = 3	*n* = 3	——	*n* = 3	*n* = 3
		Damaged	——	——	——	——	——	——	*n* = 3	——	——
	Storage	Intact	——	——	——	——	*r* = 0.179, *n* = 27	*r* = 0.224, *n* = 27	*n* = 3	——	*r* = −0.082, *n* = 18
		Damaged	——	——	——	——	*r* = 0.149, *n* = 15	*r* = −0.101, *n* = 15	*n* = 3	——	——
Aromatic compounds	Non-storage	Intact	*n* = 3	*n* = 3	*r* = 0.710 *, *n* = 18	*r* = 0.458 *, *n* = 21	*r* = 0.555 **, *n* = 36	*r* = 0.315, *n* = 33	*r* = −0.751 *, *n* = 12	*n* = 3	*n* = 3
		Damaged	——	*n* = 3	*r* = 0.554 *, *n* = 18	*r* = 0.136, *n* = 21	*r* = −0.095, *n* = 36	*r* = −0.276, *n* = 33	*n* = 3	——	——
	Storage	Intact	——	——	*r* = −0.107, *n* = 15	*r* = −0.056, *n* = 18	*r* = 0.287, *n* = 33	*r* = −0.088, *n* = 33	*n* = 6	*r* = 0.640 *, *n* = 24	*r* = 0.169, *n* = 21
		Damaged	——	——	*r* = −0.311, *n* = 15	*r* = −0.170, *n* = 15	*r* = 0.688 **, *n* = 36	*r* = 0.749 **, *n* = 36	*n* = 6	*r* = 0.271, *n* = 27	*r* = 0.220, *n* = 18

* indicates significant difference at *p* < 0.05 and ** indicates significant difference at *p* < 0.01.

## Data Availability

The data supporting the findings of this study are available from the corresponding author upon request.

## Appendix A

**Table A1:** List of the biogenic volatile organic compounds (BVOC) observed and wounding-dependent changes for monoterpenes, sesquiterpenes and aromatic compounds emitted from leaves of 45 subtropical woody species: compound name, molecular formula, emission rate ± SE.

Species	Isoprene		Monoterpenes			Sesquiterpenes			Aromatic Compounds		
	Rate (nmol m^−2^ s^−1^)		Compound	Rate (nmol m^−2^ s^−1^)		Compound	Rate (nmol m^−2^ s^−1^)		Compound	Rate (nmol m^−2^ s^−1^)	
	Intact	Injured		Intact	Injured		Intact	Injured		Intact	Injured
*Acer buergerianum*			C_10_H_16_O Camphor	0.012 ± 0.0021	0.014 ± 0.002				C_10_H_12_ 2,5-dimethylstyrene	0.059 ± 0.014	0.092 ± 0.009
			C_10_H_16_ α-pinene		0.069 ± 0.0012				C_10_H_12_O benzenebutanal		0.0015 ± 0.00021
			C_10_H_16_ Limonene		0.024 ± 0.0010						
			C_10_H_16_ *α*-phellandrene		0.012 ± 0.0042						
			C_10_H_16_ Pseudolimonene		0.011 ± 0.0011						
			C_10_H_16_ *α*-terpinolene		0.0011 ± 0.00043						
*Acer cinnamomifolium*			C_10_H_16_ *γ*-terpinene	0.0017 ± 0.00023	0.0041 ± 0.00062				C_10_H_12_ 2,5-dimethylstyrene	0.021 ± 0.0056	0.031 ± 0.0020
			C_10_H_16_ *α*-phellandrene	0.00072 ± 0.00004	* 0.0029 ± 0.0004						
			C_10_H_16_O Camphor	0.0037 ± 0.0007	0.0059 ± 0.00081						
			C_10_H_16_ (*E*)-*β*-ocimene	0.0020 ± 0.00033							
*A* *cer henryi*			C_10_H_16_ (*E*)-*β*-ocimene	0.0031 ± 0.000010	0.0032 ± 0.0013	C_15_H_24_ bicyclogermacrene	0.028 ± 0.003		C_10_H_12_ 2,5-dimethylstyrene	* 0.0038 ± 0.0009	0.00061 ± 0.00025
			C_10_H_16_ *α*-pinene	0.0018 ± 0.00020	0.0030 ± 0.00033	C_15_H_24_ *δ*-guaiene	0.024 ± 0.00034				
			C_10_H_16_ Camphor		0.011 ± 0.0018	C_15_H_24_ aciphyllene	0.015 ± 0.0013				
			C_10_H_16_ Limonene	0.0022 ± 0.00049		C_15_H_24_ *α*-muurolene	0.018 ± 0.0053				
						C_15_H_24_ *δ*-selinene	0.011 ± 0.00022				
*Acer palmatum*			C_10_H_16_ *α*-pinene	0.012 ± 0.00083	0.015 ± 0.0028	C_15_H_24_ *cis*-muurola-4(14),5-diene	0.0032 ± 0.0012		C_14_H_18_O_2_ 4-cyclopentyl ethylbenzoate	0.17 ± 0.018	
			C_10_H_16_O Camphor	0.030 ± 0.0055	0.029 ± 0.0035						
*Broussonetia papyrifera*	1.2 ± 0.051	0.81 ± 0.33	C_10_H_16_ *α*-pinene	0.0080 ± 0.0011	0.0054 ± 0.00096	C_15_H_24_ *α*-muurolene	0.011 ± 0.00001				
			C_10_H_16_O Camphor	0.019 ± 0.0051	0.0085 ± 0.0016	C_15_H_24_ bicyclogermacrene	0.0078 ± 0.00072				
*Camellia japonica*			C_10_H_16_ Limonene		0.0034 ± 0.00018				C_14_H_18_O_2_ 4-cyclopentyl ethylbenzoate	0.0032 ± 0.00063	* 0.017 ± 0.00053
			C_10_H_16_ *α*-pinene	0.0032 ± 0.00032	0.0025 ± 0.00033				C_7_H_6_O benzaldehyde	* 0.0023 ± 0.0004	0.00071 ± 0.00038
			C_10_H_16_O Camphor	0.011 ± 0.0013	0.011 ± 0.0010						
*Camptotheca acuminata*	0.064 ± 0.0062	0.051 ± 0.016	C_10_H_16_ *α*-pinene	0.00044 ± 0.00001	0.0031 ± 0.00023				C_13_H_18_O_2_ 2-methyl-3-phenylpropyl benzoate		0.00043 ± 0.0000
			C_10_H_16_O Camphor		0.0014 ± 0.00013						
			C_10_H_16_ Limonene		0.0016 ± 0.00001						
*Castanopsis sclerophylla*			C_10_H_16_ α-pinene	* 0.097 ± 0.034	0.020 ± 0.0024				C_10_H_12_ 2,5-dimethylstyrene	0.012 ± 0.0060	
			C_10_H_16_ Limonene	* 0.022 ± 0.010	0.0034 ± 0.00076				C_13_H_18_O_2_ 2,4-butylethylbenzoate		0.013 ± 0.00023
			C_10_H_16_ Cyclofeuchene	* 0.047 ± 0.015	0.0086 ± 0.00016						
			C_10_H_16_ *γ*-terpinene	* 0.046 ± 0.023	0.0079 ± 0.0020						
			C_10_H_16_ α-terpinene	0.076 ± 0.017							
			C_10_H_16_ camphene	0.041 ± 0.023							
			C_10_H_16_ Pseudolimonene	0.021 ± 0.010							
			C_10_H_16_ *α*-terpinolene	0.013 ± 0.0049							
			C_10_H_16_ (*E*)-*β*-ocimene	0.011 ± 0.0056							
			C_10_H_16_ *α*-phellandrene	0.0086 ± 0.0027							
			C_10_H_16_ *α*-fenchene	0.0060 ± 0.0028							
			C_10_H_14_ *p*-cymene	0.069 ± 0.035							
*Chimonanthus praecox*	* 0.87 ± 0.077	0.14 ± 0.44	C_10_H_16_ *α*-phellandrene	0.0064 ± 0.00023					C_14_H_18_O_2_ 2,4-butylethylbenzoate	0.0027 ± 0.00033	
			C_10_H_16_ *α*-pinene	0.0023 ± 0.00063	0.0042 ± 0.00043				C_8_H_10_ *p*-xylene	0.0015 ± 0.0004	
			C_10_H_16_ *γ*-terpinene	0.0078 ± 0.00003	* 0.024 ± 0.0033						
			C_10_H_16_ Pseudolimonene	0.0037 ± 0.00058	0.0075 ± 0.00068						
			C_10_H_16_ *β*-phellandrene	0.0025 ± 0.00022	0.0037 ± 0.00063						
			C_10_H_16_ Limonene	0.0012 ± 0.000042	* 0.0029 ± 0.0003						
			C_10_H_16_ *α*-terpinolene	0.00061 ± 0.00001	* 0.0015 ± 0.0002						
			C_10_H_16_O Camphor	0.0022 ± 0.00050	* 0.0058 ± 0.0011						
			C_10_H_14_ *p*-cymene	0.0021 ± 0.00059	* 0.010 ± 0.0034						
			C_10_H_16_ (*E*)-*β*-ocimene		0.0071 ± 0.0013						
*Cinnamomum camphora*			C_10_H_16_ (*E*)-*β*-ocimene	0.20 ± 0.085	0.37 ± 0.0093	C_15_H_24_ *γ*-humulene	0.039 ± 0.0057		C_10_H_12_ 2,5-dimethylstyrene	0.013 ± 0.0063	* 0.21 ± 0.083
			C_10_H_16_ α-pinene	0.041 ± 0.015	* 0.57 ± 0.040	C_15_H_24_ bicyclogermacrene	0.015 ± 0.0041		C_14_H_18_O_2_ 2,4-butylethylbenzoate	* 0.030 ± 0.011	0.012 ± 0.00023
			C_10_H_16_ *γ*-terpinene	0.024 ± 0.0097	* 0.27 ± 0.0056	C_15_H_24_ *β*-longipinene	0.0089 ± 0.0032		C_10_H_12_O_2_ dicyclopentadiene diepoxide		0.012 ± 0.00010
			C_10_H_16_ *α*-terpinene	0.018 ± 0.0070	* 0.30 ± 0.14	C_15_H_24_ *cis*-muurola-4(14),5-diene	0.0050 ± 0.0000				
			C_10_H_16_ Limonene	0.011 ± 0.0037	* 0.56 ± 0.040	C_15_H_24_ *δ*-selinene	0.0053 ± 0.00001	* 0.022 ± 0.0016			
			C_10_H_16_ *α*-phellandrene	0.0048 ± 0.00073	0.17 ± 0.066						
			C_10_H_16_ Pseudolimonene	0.011 ± 0.0028	* 0.18 ± 0.055						
			C_10_H_16_ *α*-terpinolene	0.0069 ± 0.0027	* 0.21 ± 0.085						
			C_10_H_16_ *α*-fenchene	0.0010 ± 0.0001	* 0.026 ± 0.0046						
			C_10_H_16_O Camphor	0.30 ± 0.000012	* 2.6 ± 0.79						
			C_10_H_18_O eucalyptol	0.16 ± 0.095	* 1.1 ± 0.39						
			C_10_H_14_ *p*-cymene	0.058 ± 0.029	* 0.49 ± 0.13						
			C_10_H_16_ camphene		0.52 ± 0.068						
			C_10_H_16_ 3-carene		0.27 ± 0.046						
			C_10_H_16_ *β*-phellandrene		0.25 ± 0.090						
			C_10_H_18_O *α*-terpineol		0.45 ± 0.13						
			C_10_H_18_O *trans*-4-thujanol		0.23 ± 0.074						
			C_10_H_18_O endo-borneol		0.099 ± 0.0081						
*Cinnamomum japonicum*	* 0.55 ± 0.071	0.16 ± 0.099	C_10_H_16_ *α*-pinene	0.0055 ± 0.0014	* 0.075 ± 0.028	C_15_H_24_ *cis*-muurola-4(14),5-diene	0.0039 ± 0.0010		C_10_H_12_ 2,5-dimethylstyrene	0.0020 ± 0.0011	0.0017 ± 0.00066
			C_10_H_16_ camphene	0.00090 ± 0.00009	* 0.030 ± 0.012	C_15_H_24_ *γ*-gurjunene	0.026 ± 0.012	0.018 ± 0.00019			
			C_10_H_14_ *p*-cymene	0.00053 ± 0.00005	* 0.0065 ± 0.0030	C_15_H_24_ *β*-copaene	0.11 ± 0.0069				
			C_10_H_16_ Limonene		0.035 ± 0.010	C_15_H_24_ caryophyllene	0.059 ± 0.000013				
			C_10_H_16_ Pseudolimonene		0.027 ± 0.0097	C_15_H_24_ bicyclogermacrene	0.055 ± 0.0013				
			C_10_H_16_ *β*-phellandrene		0.011 ± 0.0055	C_15_H_24_ *δ*-guaiene	0.033 ± 0.014				
			C_10_H_16_ *α*-phellandrene		0.011 ± 0.0039	C_15_H_24_ *γ*-humulene	0.017 ± 0.00012				
			C_10_H_16_ (*E*)-*β*-ocimene		0.0056 ± 0.0010	C_15_H_24_ *β*-maaliene	0.014 ± 0.0044				
			C_10_H_16_ *γ*-terpinene		0.0027 ± 0.00025	C_15_H_24_ *δ*-selinene	0.0052 ± 0.0017	0.0072 ± 0.00052			
			C_10_H_16_O Camphor		0.24 ± 0.073						
			C_10_H_18_O endo-Borneol		0.010 ± 0.00064						
*Cyclobalanopsis glauca*			C_10_H_16_ *α*-pinene	0.047 ± 0.0053	0.035 ± 0.016				C_10_H_12_ 3,4-dimethylstyrene	0.00093 ± 0.0002	0.00070 ± 0.00002
			C_10_H_16_ Pseudolimonene	0.0050 ± 0.0013	0.0088 ± 0.00007				C_10_H_12_O benzenebutanal	0.0012 ± 0.00009	
			C_10_H_16_ camphene	0.0026 ± 0.00022	0.0039 ± 0.00019				C_13_H_18_O_2_ 2,4-butylethylbenzoate	0.0018 ± 0.00021	
			C_10_H_16_ Limonene	0.0025 ± 0.00023	0.0032 ± 0.00086				C_8_H_10_ *p*-xylene	0.00062 ± 0.000061	
			C_10_H_16_ α-terpinolene	0.00051 ± 0.00003	* 0.0013 ± 0.0001				C_15_H_14_O_2_ 2-phenylethyl benzoate		0.00086 ± 0.00002
			C_10_H_16_O Camphor	0.013 ± 0.0015	0.016 ± 0.0024						
			C_10_H_14_ *p*-cymene	0.00073 ± 0.00022	0.00077 ± 0.0002						
*Cyclobalanopsis myrsinifolia*			C_10_H_16_ *α*-pinene	0.11 ± 0.0098	0.052 ± 0.023				C_10_H_12_ 3,4-dimethylstyrene	0.010 ± 0.00021	
			C_10_H_16_ *γ*-terpinene	* 0.050 ± 0.026	0.015 ± 0.0069				C_14_H_18_O_2_ 4-cyclopentyl ethylbenzoate	0.065 ± 0.025	0.016 ± 0.0040
			C_10_H_16_ Cyclofeuchene	0.055 ± 0.016							
			C_10_H_16_ *α*-terpinene	0.032 ± 0.16	0.016 ± 0.0061						
			C_10_H_16_ Pseudolimonene	0.027 ± 0.0066	0.016 ± 0.0039						
			C_10_H_16_ Limonene	0.015 ± 0.0040	0.011 ± 0.0038						
			C_10_H_16_ camphene	* 0.014 ± 0.0040	0.0040 ± 0.0013						
			C_10_H_16_ *α*-terpinolene	* 0.011 ± 0.0047	0.0043 ± 0.0010						
			C_10_H_16_ *β*-phellandrene	* 0.010 ± 0.0042	0.0036 ± 0.00091						
			C_10_H_16_ *α*-phellandrene	0.0065 ± 0.0028	0.0036 ± 0.00090						
			C_10_H_16_ *α*-fenchene	0.0023 ± 0.00046							
			C_10_H_16_O Camphor	0.0032 ± 0.00063	0.0024 ± 0.00017						
			C_10_H_14_ *p*-cymene	* 0.069 ± 0.032	0.025 ± 0.012						
*Elaeocarpus glabripetalus* var. *glabripetalus*	0.93 ± 0.18	0.70 ± 0.37	C_10_H_16_ *α*-pinene	0.0016 ± 0.00031	0.0025 ± 0.00022				C_13_H_18_O_2_ 2,4-butylethylbenzoate	* 0.031 ± 0.0089	0.013 ± 0.0040
			C_10_H_16_O Camphor	0.0045 ± 0.0010	0.0067 ± 0.0010				C_15_H_14_O_2_ 2-phenylethyl benzoate	0.00048 ± 0.000073	
									C_16_H_16_O_2_ 2-phenylethyl benzoate		0.00086 ± 0.00009
*Gardenia jasminoides*			C_10_H_16_O camphor	0.013 ± 0.0035	0.014 ± 0.0037				C_14_H_18_O_2_ 4-cyclopentyl ethylbenzoate	0.060 ± 0.014	0.036 ± 0.0069
			C_10_H_16_ *α*-pinene		0.0014 ± 0.00013						
*Ginkgo biloba*			C_10_H_16_ *α*-pinene	0.0038 ± 0.0005	* 0.014 ± 0.0043				C_14_H_18_O_2_ 4-cyclopentyl ethylbenzoate	0.015 ± 0.0034	* 0.040 ± 0.0084
			C_10_H_16_O Camphor		0.035 ± 0.00078						
*Ilex chinensis*			C_10_H_16_ *α*-pinene	0.0015 ± 0.00045	* 0.0081 ± 0.0016				C_11_H_14_O_2_ 2-phenylethyl benzoate		0.0031 ± 0.00076
			C_10_H_16_O Camphor	0.012 ± 0.0020	0.025 ± 0.011				C_14_H_18_O_2_ 4-cyclopentyl ethylbenzoate		0.016 ± 0.0034
*Jasminum mesnyi*			C_10_H_16_ *α*-pinene	0.0035 ± 0.00051	0.0055 ± 0.00030				C_15_H_14_O_2_ 2-phenylethyl benzoate	0.0014 ± 0.00001	
			C_10_H_16_O Camphor	0.010 ± 0.0015	0.013 ± 0.0020				C_14_H_18_O_2_ 4-cyclopentyl ethylbenzoate	0.034 ± 0.0059	0.038 ± 0.0057
*Koelreuteria bipinnata* var. *integrifoliola*			C_10_H_16_ *α*-pinene	0.0047 ± 0.00013	0.0046 ± 0.00052	C_15_H_24_ *β*-longipinene	0.36 ± 0.0019				
						C_15_H_24_ valerena-4,7(11)-diene	0.17 ± 0.010				
						C_15_H_24_ *γ*-himachalene	0.099 ± 0.00044				
						C_15_H_24_ *δ*-guaiene	0.059 ± 0.013				
						C_15_H_24_ *β*-elemene	0.056 ± 0.017				
						C_15_H_24_ *α*-guaiene	0.021 ± 0.00086				
						C_15_H_24_ bicyclogermacrene	0.017 ± 0.00063				
						C_15_H_24_ *β*-copaene	0.016 ± 0.00058				
						C_15_H_24_ aromadendrene	0.013 ± 0.000064				
*Ligustrum lucidum*			C_10_H_16_ *α*-pinene	0.0020 ± 0.00045	* 0.011 ± 0.00022				C_14_H_18_O_2_ 4-cyclopentyl ethylbenzoate	0.046 ± 0.0069	0.051 ± 0.0077
									C_9_H_10_O_2_ 2-phenylethyl benzoate		0.0036 ± 0.00025
*Liriodendron chinense*			C_10_H_16_ Pseudolimonene	0.027 ± 0.0073	* 0.12 ± 0.025	C_15_H_24_ *cis*-muurola-3,5-diene		0.0054 ± 0.00027	C_13_H_18_O_2_ 2,4-butylethylbenzoate	0.091 ± 0.022	0.093 ± 0.015
			C_10_H_16_ *α*-pinene	0.024 ± 0.0060	0.049 ± 0.018				C_10_H_12_ 3,4-dimethylstyrene		0.0041 ± 0.00063
			C_10_H_16_ Limonene	0.013 ± 0.0044	0.016 ± 0.0040						
			C_10_H_16_ *γ*-terpinene	0.0052 ± 0.0020	* 0.055 ± 0.0057						
			C_10_H_16_ *α*-fenchene	0.0022 ± 0.00064	0.0031 ± 0.00005						
			C_10_H_16_ (*E*)-*β*-ocimene		0.082 ± 0.020						
			C_10_H_16_ *α*-terpinene		0.034 ± 0.0049						
			C_10_H_16_ camphene		0.025 ± 0.00083						
			C_10_H_16_ *α*-phellandrene		0.022 ± 0.011						
			C_10_H_16_ 3-carene		0.0084 ± 0.00063						
			C_10_H_18_O eucalyptol		0.0095 ± 0.00044						
			C_10_H_14_ *p*-cymene		0.0099 ± 0.0026						
*Lithocarpus harlandii*			C_10_H_16_ (*E*)-*β*-ocimene	* 0.050 ± 0.021	0.016 ± 0.0039	C_15_H_24_ *cis*-muurola-3,5-diene	0.0030 ± 0.0000011		C_10_H_12_ 3,4-dimethylstyrene	0.0010 ± 0.00001	
			C_10_H_16_ *α*-pinene	0.012 ± 0.0051	0.0050 ± 0.00083				C_14_H_18_O_2_ 4-cyclopentyl ethylbenzoate	0.041 ± 0.010	
			C_10_H_16_O Camphor	0.0080 ± 0.00031	0.0063 ± 0.00027						
			C_10_H_16_ *α*-terpinolene	0.015 ± 0.0010							
			C_10_H_16_ Pseudolimonene	0.012 ± 0.00015							
			C_10_H_16_ *γ*-terpinene	0.010 ± 0.0039							
			C_10_H_16_ Limonene	0.0032 ± 0.000017							
			C_10_H_16_ *α*-phellandrene	0.0028 ± 0.000070							
			C_10_H_14_ *p*-cymene	0.0077 ± 0.0020							
*Machilus leptophylla*			C_10_H_16_ *α*-pinene	0.11 ± 0.0028	0.21 ± 0.028	C_15_H_24_ *β*-copaene	0.094 ± 0.00087		C_10_H_12_ 3,4-dimethylstyrene	0.019 ± 0.0059	0.022 ± 0.0014
			C_10_H_16_ camphene	0.054 ± 0.019	0.072 ± 0.013	C_15_H_24_ *cis*-muurola-4(14),5-diene	0.040 ± 0.014		C_14_H_18_O_2_ 4-cyclopentyl ethylbenzoate	0.058 ± 0.010	0.029 ± 0.0029
			C_10_H_16_ Pseudolimonene	0.036 ± 0.017	* 0.071 ± 0.013	C_15_H_24_ *σ*-selinene	0.054 ± 0.0051				
			C_10_H_16_ *α*-terpinene	0.029 ± 0.0095	0.058 ± 0.017	C_15_H_24_ *β*-maaliene	0.040 ± 0.0043				
			C_10_H_16_ Limonene	0.029 ± 0.0088	0.059 ± 0.0095	C_15_H_24_ bicyclogermacrene	0.018 ± 0.0062				
			C_10_H_16_ *γ*-terpinene	0.028 ± 0.011	0.034 ± 0.0066	C_15_H_24_ *β*-elemene	0.049 ± 0.014				
			C_10_H_16_ *β*-phellandrene	0.0086 ± 0.0010	* 0.040 ± 0.011	C_15_H_24_ aromadendrene	0.018 ± 0.0062				
			C_10_H_16_ *α*-terpinolene	0.0068 ± 0.0017	0.011 ± 0.0018	C_15_H_24_ *α*-bulnesene	0.012 ± 0.00073	0.0027 ± 0.00034			
			C_10_H_16_ *α*-phellandrene	0.0083 ± 0.0036	* 0.16 ± 0.0049	C_15_H_24_ *α*-ylangene	0.0065 ± 0.0019				
			C_10_H_16_ *α*-fenchene	0.0058 ± 0.0022	0.0076 ± 0.0011	C_15_H_24_ germacrene D	0.0030 ± 0.0010				
			C_10_H_14_ *p*-cymene	0.050 ± 0.014	0.10 ± 0.016	C_15_H_24_ *β*-longipinene	0.0066 ± 0.0019				
			C_10_H_16_ (*E*)-*β*-ocimene		0.011 ± 0.0011	C_15_H_24_ aromadendrene	0.017 ± 0.0021				
			C_10_H_16_ *β*-fenchene		0.0035 ± 0.00032	C_15_H_24_ *cis*-muurola-3,5-diene	0.041 ± 0.000011				
			C_10_H_18_O eucalyptol		0.015 ± 0.0012	C_15_H_24_ β-longipinene	0.026 ± 0.0044				
			C_10_H_16_ Cyclofeuchene	0.029 ± 0.00033	0.019 ± 0.0034	C_15_H_24_ (-)-aristolene	0.0038 ± 0.00081				
*Machilus pauhoi*			C_10_H_16_ α-pinene	0.039 ± 0.022	* 0.096 ± 0.021	C_15_H_24_ *σ*-selinene	0.055 ± 0.015		C_13_H_18_O_3_ methanol, [4-(1,1-dimethylethyl)phenoxy]-, acetate	0.0015 ± 0.00004	
			C_10_H_16_ *γ*-terpinene	0.052 ± 0.0090	0.064 ± 0.012	C_15_H_24_ (-)-aciphyllene	0.018 ± 0.0065		C_10_H_12_ *o*-cumenene	0.0045 ± 0.0011	0.0068 ± 0.0015
			C_10_H_16_ Pseudolimonene	0.017 ± 0.0023	0.020 ± 0.0063	C_15_H_24_ *β*-copaene	0.018 ± 0.0078		C_16_H_16_O_2_ 2-phenylethyl benzoate		0.019 ± 0.0071
			C_10_H_16_ α-terpinolene	0.0058 ± 0.00093	0.0066 ± 0.0016	C_15_H_24_ *α*-muurolene	0.0086 ± 0.0026				
			C_10_H_16_ *β*-phellandrene	0.0056 ± 0.000057	0.0063 ± 0.0017	C_15_H_24_ aromadendrene	0.0025 ± 0.0011				
			C_10_H_16_ Limonene	0.0056 ± 0.0024	0.012 ± 0.0028	C_15_H_24_ *β*-patchoulene	0.0055 ± 0.0011				
			C_10_H_16_ camphene	0.011 ± 0.0038	0.011 ± 0.00051	C_15_H_24_ caryophyllene	0.0030 ± 0.00060				
			C_10_H_16_ *α*-phellandrene	0.0070 ± 0.000052	0.0085 ± 0.0018						
			C_10_H_16_ (*E*)-*β*-ocimene		0.0078 ± 0.0016						
			C_10_H_14_ *p*-cymene	0.038 ± 0.015	0.021 ± 0.0078						
*Machilus thunbergii*			C_10_H_16_ α-terpinolene	0.025 ± 0.0017	0.053 ± 0.0037				C_15_H_14_O_2_ 2-phenylethyl benzoate	0.0012 ± 0.00026	
			C_10_H_16_ *γ*-terpinene	0.0039 ± 0.00023	* 0.010 ± 0.0024				C_14_H_18_O_2_ 4-cyclopentyl ethylbenzoate	0.017 ± 0.0047	* 0.094 ± 0.045
			C_10_H_16_ α-terpinene	0.0029 ± 0.000010	* 0.013 ± 0.00005				C_9_H_10_O_2_ 2-phenylethyl formate		0.0037 ± 0.00033
			C_10_H_16_ Limonene	0.00063 ± 0.00024	* 0.012 ± 0.0038						
			C_10_H_16_ Pseudolimonene		0.058 ± 0.0011						
			C_10_H_16_ camphene		0.0024 ± 0.00013						
			C_10_H_14_ *p*-cymene		0.0041 ± 0.0010						
			C_10_H_16_ α-thujene	0.011 ± 0.00083	0.0093 ± 0.00044						
			C_10_H_16_O Camphor	0.023 ± 0.0046	0.021 ± 0.0054						
*Magnolia denudata*			C_10_H_16_ *α*-pinene	0.0013 ± 0.00025	0.0013 ± 0.00007				C_14_H_18_O_2_ 4-cyclopentyl ethylbenzoate	0.046 ± 0.018	0.038 ± 0.013
			C_10_H_16_O Camphor	0.0017 ± 0.00020	0.0020 ± 0.00023				C_15_H_14_O_2_ 2-phenylethyl benzoate		0.0010 ± 0.000071
			C_10_H_18_O eucalyptol	0.0092 ± 0.00034							
*Magnolia grandiflora*			C_10_H_16_ camphene	0.0028 ± 0.000051	0.0071 ± 0.0014	C_15_H_24_ *β*-elemene	0.0078 ± 0.0044	* 0.081 ± 0.0076	C_10_H_12_ 3,4-dimethylstyrene	0.00066 ± 0.0002	* 0.0023 ± 0.00090
			C_10_H_16_ Pseudolimonene	0.0026 ± 0.00053	* 0.011 ± 0.0041	C_15_H_24_ *β*-copaene		0.0040 ± 0.0007	C_12_H_16_O 1-(4-methylphenyl)-1-Pentanone		0.0033 ± 0.0012
			C_10_H_16_ Limonene	0.0014 ± 0.0003	* 0.0064 ± 0.0016	C_15_H_24_ *cis*-muurola-4(14),5-diene	0.028 ± 0.0079				
			C_10_H_16_ *γ*-terpinene	0.0013 ± 0.0002	* 0.0048 ± 0.0009	C_15_H_24_ caryophyllene	0.023 ± 0.00001				
			C_10_H_16_ *α*-terpinene	0.00093 ± 0.00006	* 0.0032 ± 0.0005	C_15_H_24_ bicyclogermacrene	* 0.015 ± 0.0041	0.0014 ± 0.0005			
			C_10_H_14_ *p*-cymene	0.0021 ± 0.0005	0.0031 ± 0.0012	C_15_H_24_ *γ*-gurjunene	0.015 ± 0.0062	0.0087 ± 0.0028			
			C_10_H_16_ *α*-fenchene		0.0014 ± 0.0002						
			C_10_H_16_O Camphor		0.058 ± 0.0311						
			C_10_H_16_ α-pinene	0.042 ± 0.0069	0.040 ± 0.016						
			C_10_H_16_ *α*-thujene	0.0025 ± 0.0002							
*Magnolia liliflora*			C_10_H_16_ *α*-pinene	0.076 ± 0.032	0.13 ± 0.018	C_15_H_24_ *γ*-gurjunene	0.14 ± 0.038		C_14_H_18_O_2_ 2-phenylethyl benzoate	0.013 ± 0.0012	0.014 ± 0.0031
			C_10_H_16_ Pseudolimonene	0.063 ± 0.035	* 0.18 ± 0.019	C_15_H_24_ *γ*-humulene	0.040 ± 0.0075		C_10_H_12_ 3,4-dimethylstyrene	0.0036 ± 0.0014	0.0037 ± 0.0006
			C_10_H_16_ *γ*-terpinene	0.035 ± 0.016	* 0.14 ± 0.031	C_15_H_24_ *β*-longipinene	0.11 ± 0.0002		C_9_H_12_O 3,4-dimethylbenzyl alcohol	0.011 ± 0.0033	
			C_10_H_16_ Limonene	0.027 ± 0.011	* 0.068 ± 0.0098	C_15_H_24_ *δ*-guaiene	0.016 ± 0.0037				
			C_10_H_16_ camphene	0.025 ± 0.011	0.040 ± 0.0061	C_15_H_24_ germacrene D	0.012 ± 0.0031				
			C_10_H_16_ (*E*)-*β*-ocimene	0.0035 ± 0.0004	* 0.011 ± 0.0034	C_15_H_24_ *β*-bourbonene		0.0032 ± 0.0004			
			C_10_H_16_ α-terpinolene	0.0029 ± 0.0003	0.0044 ± 0.0008						
			C_10_H_16_ *α*-thujene	0.0013 ± 0.00006	0.0025 ± 0.0004						
			C_10_H_16_ *β*-phellandrene	0.00063 ± 0.0001	0.0014 ± 0.0003						
			C_10_H_18_O eucalyptol	0.012 ± 0.0019	0.020 ± 0.0019						
			C_10_H_14_ *p*-cymene	0.047 ± 0.022	* 0.13 ± 0.017						
			C_10_H_16_ α-terpinene		0.022 ± 0.0052						
			C_10_H_16_O Camphor		0.13 ± 0.042						
			C_10_H_18_O *α*-terpineol		0.0090 ± 0.0003						
*Melia azedarach*			C_10_H_16_ *α*-pinene	0.00052 ± 0.00009	0.00094 ± 0.0002				C_8_H_10_ *p*-xylene	0.0032 ± 0.0002	
			C_10_H_16_O camphor	0.00062 ± 0.00001					C_10_H_18_O_2_ cis-3-Hexenyl iso-butyrate		0.00093 ± 0.00005
									C_15_H_14_O_2_ 2-phenylethyl benzoate		0.0014 ± 0.0030
*Michelia chapensis*			C_10_H_16_ *α*-pinene	0.090 ± 0.031	0.11 ± 0.027				C_10_H_12_ 3,4-dimethylstyrene	0.013 ± 0.0050	* 0.049 ± 0.021
			C_10_H_16_ *α*-terpinene	0.061 ± 0.029	0.11 ± 0.042				C_9_H_12_O 3,4-dimethylbenzyl alcohol		0.082 ± 0.032
			C_10_H_16_ camphene	0.054 ± 0.017	0.055 ± 0.023				C_9_H_10_O_2_ (2-methylphenyl)methyl formate		0.0031 ± 0.0003
			C_10_H_16_ Limonene	0.041 ± 0.019	0.061 ± 0.027						
			C_10_H_16_ *α*-thujene	0.029 ± 0.018	* 0.11 ± 0.031						
			C_10_H_16_ Pseudolimonene	0.027 ± 0.013	0.052 ± 0.025						
			C_10_H_16_ *γ*-terpinene	0.018 ± 0.0068	* 0.13 ± 0.048						
			C_10_H_16_ *β*-phellandrene	0.013 ± 0.0045	* 0.038 ± 0.016						
			C_10_H_16_ *α*-phellandrene	0.0087 ± 0.0043	0.015 ± 0.0093						
			C_10_H_16_ *α*-fenchene	0.0066 ± 0.0021	0.0087 ± 0.0036						
			C_10_H_16_ *α*-terpinolene	0.0036 ± 0.0013	* 0.040 ± 0.015						
			C_10_H_18_O eucalyptol	0.031 ± 0.0096	0.037 ± 0.014						
			C_10_H_14_ *p*-cymene	0.10 ± 0.028	* 0.20 ± 0.033						
			C_10_H_16_ 3-carene		0.015 ± 0.0026						
			C_10_H_18_O *trans*-4-thujanol		0.010 ± 0.0011						
*Michelia figo*			C_10_H_16_ *α*-pinene	0.00043 ± 0.00008	* 0.0039 ± 0.0003	C_15_H_24_ germacrene D	0.0052 ± 0.00001		C_11_H_14_O_2_ 2-phenylethyl benzoate		0.0011 ± 0.00041
			C_10_H_16_ camphene		0.013 ± 0.0014	C_15_H_24_ *cis*-muurola-4(14),5-diene	0.0023 ± 0.0002				
			C_10_H_16_ Limonene		0.00076 ± 0.0003						
*Michelia foveolata*			C_10_H_16_ α-thujene	0.039 ± 0.000022	0.068 ± 0.012	C_15_H_24_ *β*-copaene	0.036 ± 0.000011		C_14_H_18_O_2_ 4-cyclopentyl ethylbenzoate	* 0.14 ± 0.0087	0.064 ± 0.023
			C_10_H_16_ *γ*-terpinene	0.068 ± 0.0052	0.11 ± 0.024	C_15_H_24_ *γ*-elemene	0.034 ± 0.0056		C_10_H_12_ 2,5-dimethylstyrene	0.010 ± 0.0015	0.0096 ± 0.0054
			C_10_H_16_ *α*-terpinene	0.028 ± 0.0049	0.046 ± 0.0190	C_15_H_24_ bicyclogermacrene	0.026 ± 0.0010		C_9_H_12_O 3,4-dimethylbenzyl alcohol	0.027 ± 0.0028	
			C_10_H_16_ Limonene	0.025 ± 0.0028	0.029 ± 0.017	C_15_H_24_ *α*-muurolene	0.021 ± 0.0060		C_9_H_10_O_2_ 2-phenylethyl benzoate		0.0021 ± 0.00022
			C_10_H_16_ camphene	0.020 ± 0.0002	* 0.055 ± 0.014	C_15_H_24_ *α*-copaene	0.017 ± 0.0033				
			C_10_H_16_ *α*-terpinolene	0.0073 ± 0.0012	0.016 ± 0.0078	C_15_H_24_ -guaiene	0.014 ± 0.00041				
			C_10_H_16_ α-phellandrene	0.0062 ± 0.0013	0.019 ± 0.0035	C_15_H_24_ (-)-aristolene	0.0097 ± 0.0022				
			C_10_H_16_ *β*-phellandrene	0.0060 ± 0.0008	* 0.020 ± 0.0043	C_15_H_24_ *β*-longipinene	0.0084 ± 0.00008				
			C_10_H_16_ *α*-fenchene	0.0013 ± 0.00002	* 0.0080 ± 0.0019	C_15_H_24_ *γ*-humulene	0.0065 ± 0.00004				
			C_10_H_18_O eucalyptol	0.0070 ± 0.0005	0.020 ± 0.0030	C_15_H_24_ *β*-maaliene	0.0045 ± 0.00005				
			C_10_H_16_O Camphor		0.012 ± 0.0020	C_15_H_24_ isoledene	0.0029 ± 0.00052				
			C_10_H_14_ *p*-cymene	0.055 ± 0.0097	0.040 ± 0.020						
			C_10_H_16_ α-pinene	0.14 ± 0.019	0.082 ± 0.029						
			C_10_H_16_ (*E*)-*β*-ocimene	0.084 ± 0.0052	0.049 ± 0.023						
*Michelia maudiae*			C_10_H_16_ camphene	0.18 ± 0.041	* 1.0 ± 0.19				C_10_H_12_ 2,5-dimethylstyrene	0.018 ± 0.054	* 0.15 ± 0.027
			C_10_H_16_ α-pinene	0.12 ± 0.031	* 0.45 ± 0.079				C_13_H_18_O_2_ 2,4-butylethylbenzoate	0.031 ± 0.0042	
			C_10_H_16_ (*E*)-*β*-ocimene	0.11 ± 0.037	0.12 ± 0.027				C_9_H_12_O 3,4-dimethylbenzyl alcohol		0.077 ± 0.029
			C_10_H_16_ Cyclofeuchene	0.10 ± 0.023	* 0.32 ± 0.021						
			C_10_H_16_ Limonene	0.086 ± 0.032	* 0.43 ± 0.090						
			C_10_H_16_ Pseudolimonene	0.082 ± 0.028	* 0.44 ± 0.052						
			C_10_H_16_ *γ*-terpinene	0.036 ± 0.015	* 0.17 ± 0.045						
			C_10_H_16_ *α*-terpinene	0.028 ± 0.0095	* 0.15 ± 0.051						
			C_10_H_16_ *α*-thujene	0.019 ± 0.0074	* 0.10 ± 0.037						
			C_10_H_16_ *α*-terpinolene	0.013 ± 0.0039	* 0.16 ± 0.054						
			C_10_H_16_ *α*-phellandrene	0.0051 ± 0.00023	* 0.11 ± 0.0092						
			C_10_H_16_ *β*-phellandrene	0.0043 ± 0.0013	* 0.076 ± 0.020						
			C_10_H_18_O eucalyptol	0.023 ± 0.0093	* 0.23 ± 0.068						
			C_10_H_16_O Camphor	0.0017 ± 0.00072	* 0.21 ± 0.030						
			C_10_H_14_ *p*-cymene	0.083 ± 0.031	* 0.23 ± 0.057						
			C_10_H_16_ *β*-fenchene		0.17 ± 0.11						
			C_10_H_16_ 3-carene		0.12 ± 0.0048						
			C_10_H_18_O Fenchol		0.053 ± 0.0017						
			C_10_H_18_O endo-borneol		0.050 ± 0.0017						
			C_10_H_18_O *trans*-4-thujanol		0.024 ± 0.0.0041						
			C_10_H_18_O *α*-terpineol		0.054 ± 0.016						
*Osmanthus fragrans*			C_10_H_16_O Camphor		0.024 ± 0.000090	C_15_H_24_ *β*-longipinene	0.13 ± 0.021		C_14_H_18_O_2_ 4-cyclopentyl ethylbenzoate	* 0.017 ± 0.0038	0.0064 ± 0.00054
			C_10_H_16_ *α*-pinene		0.0049 ± 0.00051				C_10_H_12_ 3,4-dimethylstyrene	* 0.14 ± 0.039	0.029 ± 0.0069
			C_10_H_16_ Pseudolimonene		0.0099 ± 0.0014						
			C_10_H_16_ camphene	* 0.55 ± 0.047	0.20 ± 0.059						
			C_10_H_16_ Cyclofeuchene	* 0.32 ± 0.051	0.13 ± 0.00001						
			C_10_H_16_ Limonene	* 0.28 ± 0.00013	0.044 ± 0.0077						
			C_10_H_16_ *α*-terpinolene	* 0.020 ± 0.00014	0.0041 ± 0.0010						
			C_10_H_14_ *p*-cymene	* 0.36 ± 0.11	0.14 ± 0.058						
			C_10_H_16_ bornylene	0.031 ± 0.0090							
			C_10_H_16_ *α*-fenchene	0.020 ± 0.000013							
*Parakmeria lotungensis*			C_10_H_16_ α-pinene	0.028 ± 0.012	* 0.45 ± 0.16	C_15_H_24_ *β*-longipinene	0.17 ± 0.036		C_10_H_12_ 3,4-dimethylstyrene	0.012 ± 0.000014	* 0.14 ± 0.037
			C_10_H_16_ Limonene	0.021 ± 0.0077	* 0.47 ± 0.21	C_15_H_24_ *α*-copaene	0.0040 ± 0.00011		C_14_H_18_O_2_ 4-cyclopentyl ethylbenzoate	0.0029 ± 0.00020	
			C_10_H_16_ Pseudolimonene	0.019 ± 0.0099	* 0.58 ± 0.19				C_9_H_12_O *p*-cumenol		0.022 ± 0.0048
			C_10_H_16_ *β*-phellandrene	0.0020 ± 0.00051	* 0.14 ± 0.052						
			C_10_H_18_O eucalyptol	0.0057 ± 0.00062	* 0.28 ± 0.051						
			C_10_H_16_ camphene		0.46 ± 0.11						
			C_10_H_16_ α-terpinene		0.54 ± 0.19						
			C_10_H_16_ *γ*-terpinene		0.32 ± 0.094						
			C_10_H_16_ *α*-fenchene		0.17 ± 0.047						
			C_10_H_16_ (*E*)-*β*-ocimene		0.10 ± 0.041						
			C_10_H_16_ 3-carene		0.18 ± 0.059						
			C_10_H_16_ Cyclofeuchene		0.18 ± 0.061						
			C_10_H_16_ *α*-phellandrene		0.15 ± 0.059						
			C_10_H_16_ *β*-fenchene		0.068 ± 0.024						
			C_10_H_14_ *p*-cymene		0.41 ± 0.084						
			C_10_H_16_O Camphor		0.12 ± 0.047						
			C_10_H_18_O endo-borneol		0.10 ± 0.022						
			C_10_H_18_O *trans*-4-thujanol		0.082 ± 0.017						
			C_10_H_18_O Fenchol		0.064 ± 0.021						
			C_10_H_18_O *α*-terpineol		0.034 ± 0.0082						
			C_10_H_18_O Linalool		0.29 ± 0.16						
*Photinia × fraseri*			C_10_H_16_ *α*-pinene	0.0056 ± 0.00080	0.0068 ± 0.0010				C_7_H_6_O benzaldehyde	0.0018 ± 0.00042	* 0.016 ± 0.0047
			C_10_H_16_O Camphor	0.019 ± 0.0023	0.021 ± 0.0016				C_11_H_14_O_2_ 2-phenylethyl benzoate	0.0042 ± 0.00071	0.0038 ± 0.00070
			C_10_H_16_ (*E*)-*β*-ocimene		0.0068 ± 0.0010				C_8_H_10_ *p*-xylene		0.013 ± 0.0034
*Photinia serrulata*			C_10_H_16_ Limonene	0.00073 ± 0.00005	0.00091 ± 0.0002				C_14_H_18_O_2_ 4-cyclopentyl ethylbenzoate	0.015 ± 0.0054	0.016 ± 0.0020
			C_10_H_16_O Camphor	0.0032 ± 0.0010	0.0042 ± 0.00065				C_15_H_14_O_2_ 2-phenylethyl benzoate	0.00033 ± 0.000022	* 0.00081 ± 0.000041
			C_10_H_16_ *α*-pinene	0.0044 ± 0.0021	0.0032 ± 0.00071				C_7_H_6_O benzaldehyde		0.21 ± 0.042
									C_7_H_8_O benzyl alcohol		0.050 ± 0.0081
*Pittosporum tobira*			C_10_H_16_ camphene	0.079 ± 0.012	* 0.32 ± 0.082	C_15_H_24_ thujopsene	0.044 ± 0.0026		C_10_H_12_ 2,5-dimethylstyrene	0.065 ± 0.0097	* 0.17 ± 0.043
			C_10_H_16_ Cyclofeuchene	0.025 ± 0.0040	* 0.22 ± 0.012	C_15_H_24_ *β*-longipinene	0.14 ± 0.0020		C_14_H_18_O_2_ 4-cyclopentyl ethylbenzoate	0.018 ± 0.0023	
			C_10_H_16_ *α*-pinene	0.039 ± 0.022	* 0.93 ± 0.25	C_15_H_24_ *cis*-thujopsene	0.049 ± 0.0036		C_9_H_12_O 3,4-dimethylbenzyl alcohol		0.090 ± 0.0016
			C_10_H_16_ bornylene	0.0053 ± 0.00061	* 0.10 ± 0.022	C_15_H_24_ bicyclogermacrene	0.0043 ± 0.0001				
			C_10_H_16_ *β*-fenchene	0.0038 ± 0.00064	* 0.47 ± 0.19	C_15_H_24_ *γ*-amorphene	0.051 ± 0.0125				
			C_10_H_16_ Limonene	0.021 ± 0.0047	* 0.18 ± 0.048	C_15_H_24_ *γ*-cadinene	0.036 ± 0.00030				
			C_10_H_16_ *α*-fenchene		0.12 ± 0.034	C_15_H_24_ *cis*-muurola-4(14),5-diene	0.012 ± 0.0028				
			C_10_H_16_ *α*-terpinene		0.12 ± 0.041	C_15_H_24_ *γ*-elemene	0.013 ± 0.0028				
			C_10_H_16_ Pseudolimonene		0.13 ± 0.029	C_15_H_24_ *β*-copaene	0.17 ± 0.016				
			C_10_H_16_ *α*-terpinolene		0.12 ± 0.033	C_15_H_24_ *cis*-muurola-3,5-diene	* 0.057 ± 0.0074	0.017 ± 0.0070			
			C_10_H_16_ *γ*-terpinene		0.075 ± 0.030	C_15_H_24_ isoledene	0.035 ± 0.016	0.028 ± 0.018			
			C_10_H_16_ *α*-phellandrene		0.050 ± 0.013						
			C_10_H_16_ *β*-phellandrene		0.030 ± 0.0058						
			C_10_H_16_ 3-carene		0.12 ± 0.00050						
			C_10_H_16_ (*E*)-*β*-ocimene		0.0044 ± 0.0017						
			C_10_H_18_O Fenchol		0.049 ± 0.016						
			C_10_H_18_O endo-borneol		0.049 ± 0.0091						
			C_10_H_16_O Camphor		0.025 ± 0.00051						
			C_10_H_18_O fenchone		0.015 ± 0.0015						
			C_10_H_14_ *p*-cymene	0.21 ± 0.012							
*Platanus orientalis*	0.13 ± 0.044	* 0.37 ± 0.090	C_10_H_16_ α-pinene		0.0018 ± 0.00001				C_13_H_18_O_2_ 2,4-butylethylbenzoate	0.064 ± 0.030	0.13 ± 0.057
			C_10_H_16_O Camphor		0.0085 ± 0.00062						
*Prunus mume*			C_10_H_16_ α-pinene	0.0032 ± 0.00020	0.0048 ± 0.00063				C_15_H_14_O_2_ 2-phenylethyl benzoate		0.0021 ± 0.000024
			C_10_H_16_O Camphor	0.0086 ± 0.0010	0.011 ± 0.0018						
*Pyracantha fortuneana*			C_10_H_16_ camphene	0.011 ± 0.0044	0.012 ± 0.0018				C_13_H_18_O_2_ 2,4-butylethylbenzoate	0.072 ± 0.015	0.064 ± 0.014
			C_10_H_16_ (*E*)-*β*-ocimene	0.13 ± 0.00033					C_16_H_16_O_2_ 2-phenylethyl benzoate	0.0034 ± 0.0013	
			C_10_H_16_ *α*-pinene	0.017 ± 0.0061	0.015 ± 0.0015						
			C_10_H_16_O Camphor	0.012 ± 0.0050	0.010 ± 0.0013						
*Quercus serrata* var. *brevipetiolata*	1.4 ± 0.3024	1.3 ± 0.5391	C_10_H_16_ α-pinene	0.0074 ± 0.00060	* 0.023 ± 0.0010				C_14_H_18_O_2_ 4-cyclopentyl ethylbenzoate	0.019 ± 0.0064	0.023 ± 0.0026
			C_10_H_16_ Limonene	0.0019 ± 0.00027	* 0.0050 ± 0.0013						
			C_10_H_16_O Camphor	0.013 ± 0.00073	0.023 ± 0.00066						
			C_10_H_16_ Sabinene		0.0020 ± 0.00090						
			C_10_H_16_ Pseudolimonene		0.0018 ± 0.00006						
*Sapindus mukorossi*			C_10_H_16_ α-pinene	0.0020 ± 0.00011	0.0034 ± 0.00070				C_13_H_18_O_2_ 2,4-butylethylbenzoate	0.012 ± 0.0031	0.0057 ± 0.0015
			C_10_H_16_ Limonene		0.00053 ± 0.0001				C_10_H_18_O_2_ 4-hexen-1 butylethylbenzoate		0.00063 ± 0.00013
			C_10_H_16_O Camphor	0.023 ± 0.0024	0.021 ± 0.0061						
*Sophora japonica*	1.7 ± 0.29	0.76 ± 0.29	C_10_H_14_ *p*-cymene		0.00084 ± 0.0002	C_15_H_24_ *cis*-muurola-4(14),5-diene	0.00063 ± 0.000023				
*Sophora japonica* var. *japonica* f. *pendula*	0.87 ± 0.22	0.49 ± 0.21	C_10_H_16_ Cyclofeuchene	* 0.049 ± 0.00013	0.012 ± 0.0020	C_15_H_24_ *α*-muurolene	0.1498 ± 0.0024				
			C_10_H_16_ α-pinene	0.0049 ± 0.0026	0.0054 ± 0.00011	C_15_H_24_ *cis*-muurola-4(14),5-diene	0.089 ± 0.040				
			C_10_H_16_O Camphor	0.016 ± 0.0056	0.028 ± 0.00053	C_15_H_24_ *β*-copaene	0.060 ± 0.000021				
						C_15_H_24_ *σ*-cadinene	0.0356 ± 0.0080				

* indicates significant differences between intact and injured leaf blades as assessed by paired-samples *t*-tests (*p* < 0.05). All reported values are the averages of three independent replicates per species. The emission rates were averaged for each plant individual (two replicate samples taken from each species), and then the species average (three replicate specimens) was calculated.

## Appendix B

**Table A2:** Detailed list of calculated retention indices of target compounds detected in the analysis. The *t_R_*_x_, *t_R_*_n_, and *t_R_*_n+1_ in the table are the retention times of the measured compounds and the peaks of normal alkanes with carbon atoms between n and n + 1 (*t_R_*_n_ < *t_R_*_x_ < *t_R_*_n+1_).

Compounds	Molecular Formula	t_x_/min	Carbon Number (n)	t_n_/min	t_n+1_/min	Retention Index (RI)
benzyl alcohol	C_7_H_8_O	20.872	C8	25.521	29.855	692.7319
*p*-xylene	C_8_H_10_	29.507	C8	25.521	29.855	891.9705
2-phenylethyl benzoate	C_11_H_14_O_2_	30.714	C9	29.855	34.8	917.3711
2-phenylethyl benzoate	C_15_H_14_O_2_	30.726	C9	29.855	34.8	917.6138
2-phenylethyl benzoate	C_9_H_10_O_2_	30.735	C9	29.855	34.8	917.7958
cyclofeuchene	C_10_H_16_	31.294	C9	29.855	34.8	929.1001
α-pinene	C_10_H_16_	31.695	C9	29.855	34.8	937.2093
*α*-bulnesene	C_15_H_24_	32.283	C9	29.855	34.8	949.1001
*α*-fenchene	C_10_H_16_	32.425	C9	29.855	34.8	951.9717
camphene	C_10_H_16_	32.538	C9	29.855	34.8	954.2568
thujopsene	C_15_H_24_	33.098	C9	29.855	34.8	965.5814
4-cyclopentyl ethylbenzoate	C_14_H_18_O_2_	33.532	C9	29.855	34.8	974.3579
2,4-butylethylbenzoate	C_13_H_18_O_2_	33.561	C9	29.855	34.8	974.9444
pseudolimonene	C_10_H_16_	33.804	C9	29.855	34.8	979.8584
*β*-longipinene	C_15_H_24_	34.35	C9	29.855	34.8	990.8999
aciphyllene	C_15_H_24_	34.722	C9	29.855	34.8	998.4226
*α*-phellandrene	C_10_H_16_	34.809	C10	38.719	34.8	1000.2297
benzaldehyde	C_7_H_6_O	35.052	C10	38.719	34.8	1006.4302
3,4-dimethylbenzyl alcohol	C_9_H_12_O	35.399	C10	38.719	34.8	1015.2845
limonene	C_10_H_16_	35.912	C10	38.719	34.8	1028.3746
*p*-cymene	C_10_H_14_	36.137	C10	38.719	34.8	1034.1158
*β*-phellandrene	C_10_H_16_	36.309	C10	38.719	34.8	1038.5047
(*E*)-*β*-ocimene	C_10_H_16_	36.451	C10	38.719	34.8	1042.1281
eucalyptol	C_10_H_18_O	36.68	C10	38.719	34.8	1047.9714
3-carene	C_10_H_16_	37.089	C10	38.719	34.8	1058.4078
*γ*-terpinene	C_10_H_16_	37.319	C10	38.719	34.8	1064.2766
aromadendrene	C_15_H_24_	37.745	C11	42.954	38.719	1077.0012
*α*-terpinolene	C_10_H_16_	38.885	C11	42.954	38.719	1103.9197
*p*-cumenol	C_9_H_12_O	38.885	C11	42.954	38.719	1103.9197
*γ*-humulene	C_15_H_24_	39.052	C11	42.954	38.719	1107.8630
*γ*-himachalene	C_15_H_24_	39.086	C11	42.954	38.719	1108.6659
(-)-aristolene	C_15_H_24_	39.327	C11	42.954	38.719	1114.3566
2,5-dimethylstyrene	C_10_H_12_	39.433	C11	42.954	38.719	1116.8595
*β*-maaliene	C_15_H_24_	40.158	C11	42.954	38.719	1133.9787
*α*-ylangene	C_15_H_24_	41.436	C11	42.954	38.719	1164.1558
*β*-copaene	C_15_H_24_	41.736	C11	42.954	38.719	1171.2397
*δ*-selinene	C_15_H_24_	41.828	C11	42.954	38.719	1173.4120
*cis*-muurola-3,5-diene	C_15_H_24_	41.837	C11	42.954	38.719	1173.6246
germacrene D	C_15_H_24_	42.338	C11	42.954	38.719	1185.4545
*cis*-muurola-4(14),5-diene	C_15_H_24_	42.392	C11	42.954	38.719	1186.7296
isoledene	C_15_H_24_	42.66	C11	42.954	38.719	1193.0579
valerena-4,7(11)-diene	C_15_H_24_	42.78	C11	42.954	38.719	1195.8914
4-hexen-1-butylethylbenzoate	C_10_H_18_O_2_	42.868	C11	42.954	38.719	1197.9693
*β*-bourbonene	C_15_H_24_	43.123	C12	44.8	42.954	1209.1549
*β*-elemene	C_15_H_24_	43.511	C12	44.8	42.954	1230.1733
camphor	C_10_H_16_O	43.515	C12	44.8	42.954	1230.3900
bicyclogermacrene	C_15_H_24_	44.246	C12	44.8	42.954	1269.9892
*α*-muurolene	C_15_H_24_	44.262	C12	44.8	42.954	1270.8559
endo-borneol	C_10_H_18_O	44.308	C12	44.8	42.954	1273.3478
caryophyllene	C_15_H_24_	44.492	C12	44.8	42.954	1283.3153
*α*-terpineol	C_10_H_18_O	44.731	C12	44.8	42.954	1296.2622
